# In silico target fishing and pharmacological profiling for the isoquinoline alkaloids of *Macleayacordata* (*Bo Luo Hui*)

**DOI:** 10.1186/s13020-015-0067-4

**Published:** 2015-12-17

**Authors:** Qifang Lei, Haibo Liu, Yong Peng, Peigen Xiao

**Affiliations:** Institute of Medicinal Plant Development, Chinese Academy of Medical Sciences, Beijing, 100193 China

## Abstract

**Background:**

Some isoquinoline alkaloids from *Macleaya cordata* (Willd). R. Br. (*Bo Luo Hui*) exhibited antibacterial, antiparasitic, antitumor, and analgesic effects. The targets of these isoquinoline alkaloids are undefined. This study aims to investigate the compound–target interaction network and potential pharmacological actions of isoquinoline alkaloids of *M. cordata* by reverse pharmacophore database screening.

**Methods:**

The targets of 26 isoquinoline alkaloids identified from *M. cordata* were predicted by a pharmacophore-based target fishing approach. Discovery Studio 3.5 and two pharmacophore databases (PharmaDB and HypoDB) were employed for the target profiling. A compound–target interaction network of *M. cordata* was constructed and analyzed by Cytoscape 3.0.

**Results:**

Thirteen of the 65 predicted targets identified by PharmaDB were confirmed as targets by HypoDB screening. The targets in the interaction network of *M. cordata* were involved in cancer (31 targets), microorganisms (12 targets), neurodegeneration (10 targets), inflammation and autoimmunity (8 targets), parasitosis (5 targets), injury (4 targets), and pain (3 targets). Dihydrochelerythrine (**C6**) was found to hit 23 fitting targets. Macrophage migration inhibitory factor (MIF) hits 15 alkaloids (**C1**–**2**, **C11**–**16**, **C19**–**25**) was the most promising target related to cancer.

**Conclusion:**

Through in silico target fishing, the anticancer, anti-inflammatory, and analgesic effects of *M. cordata* were the most significant among many possible activities. The possible anticancer effects were mainly contributed by the isoquinoline alkaloids as active components.

## Background

**Fig. 1:**
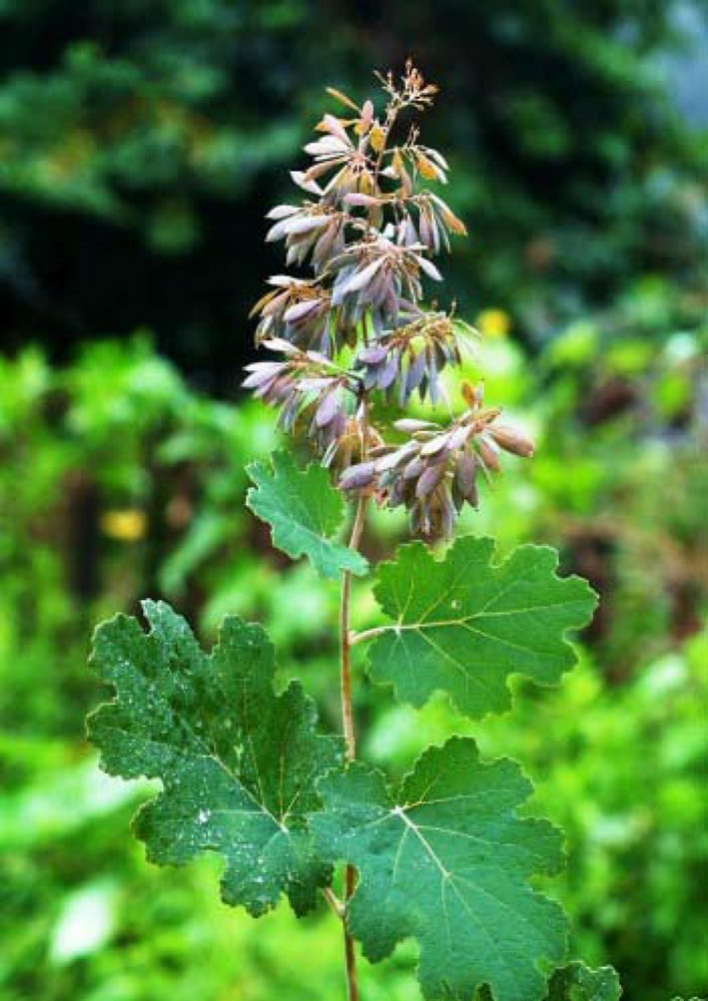
The original plant of *Macleaya cordata*

**Fig. 2:**
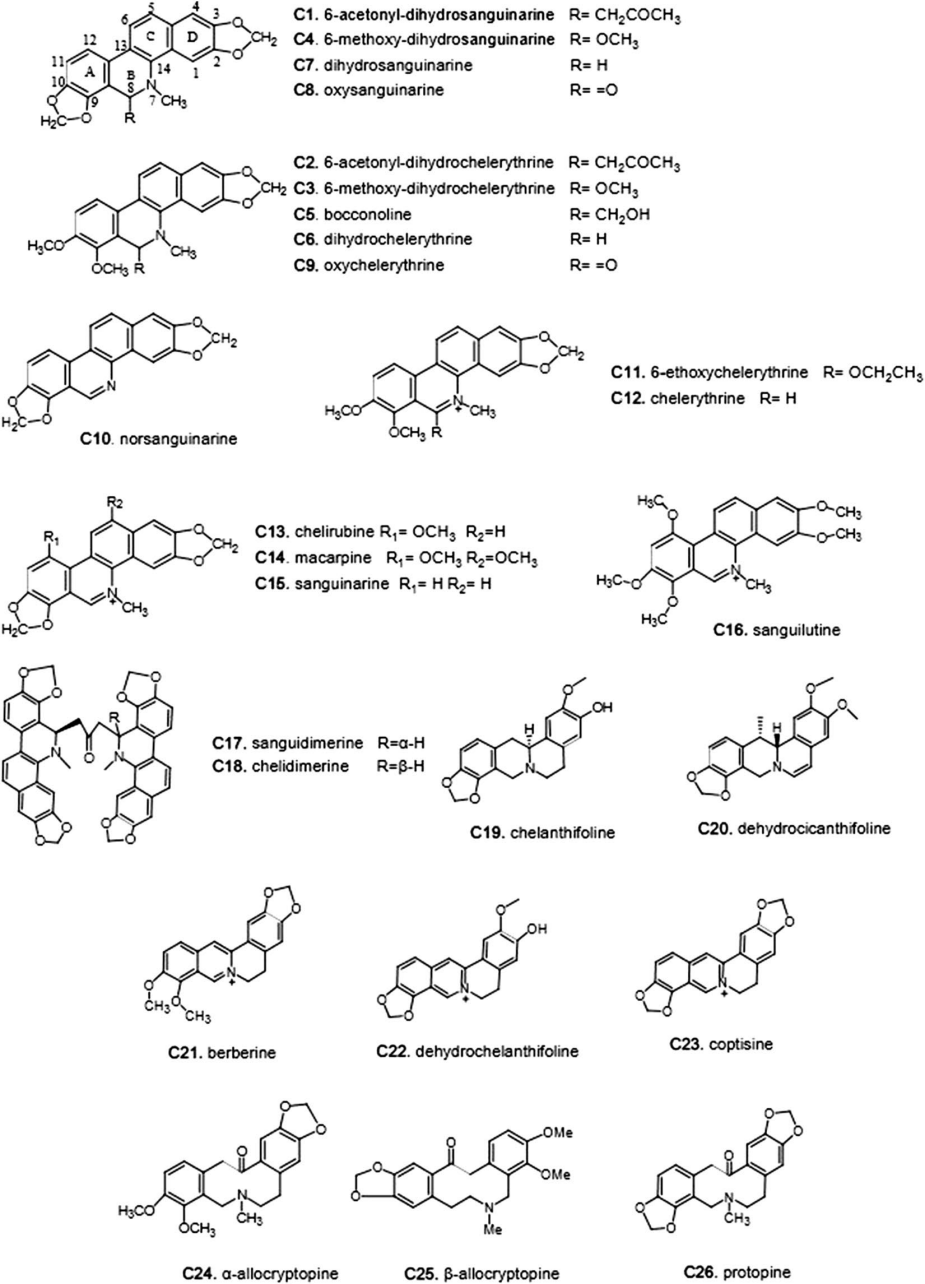
The isoquinoline alkaloids of *Macleaya cordata*

*Macleaya cordata* (Willd). R. Br. (*Bo Luo Hui*) (Fig. 1) has been used for the treatment of cancer [1], insect bites [2], and ringworm infection [3] in Mainland China, North America, and Europe. Phytochemical and pharmacological studies demonstrated that the isoquinoline alkaloids derived from *M. cordata* are its major active components [4]. Thirty isoquinoline alkaloids have been isolated from *M. cordata* (Fig. 2), including chelerythrine (**C12**), sanguinarine (**C15**), sanguidimerine (**C17**), chelidimerine (**C18**), berberine (**C21**), coptisine (**C23**), allocryptopine (**C24**, **C25**), and protopine (**C26**). These alkaloids exhibited a broad spectrum of biological activities, such as antitumor [5–8], anti-inflammatory [9–11], antimicrobial [12–14], analgesic [15], and antioxidant [16] activities.

**Fig. 3:**
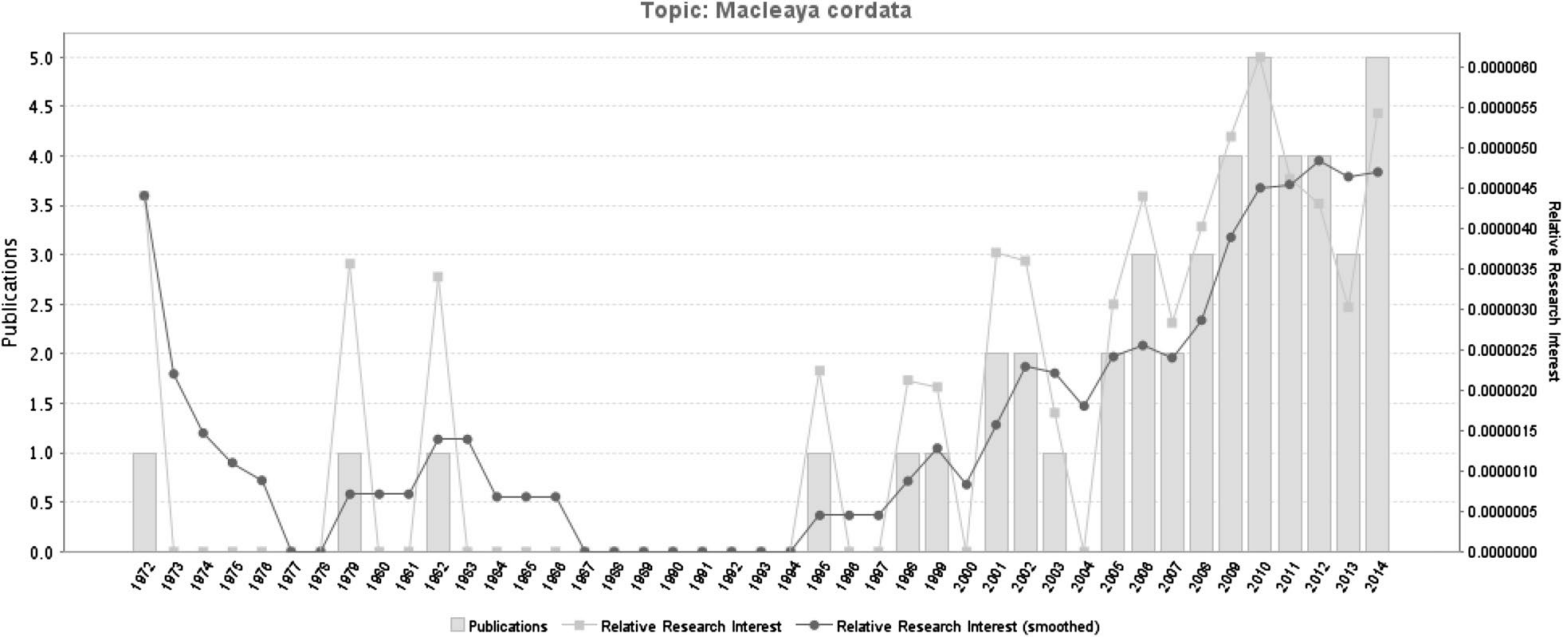
The statistics of Pubmed publications on *Macleaya cordata* between 1972 and 2014

In our previous study [17], we found that *M. cordata* could be counted not only as one of the richest resources in Mainland China among all species of the tribe Chelidonieae, but also as one of the most promising natural resources for drug discovery. *M. cordata* has gained the attention of pharmacognosists since early 1990s (Fig. 3). However, its obscure molecular actions have hindered its use in drug development.

Although protein–ligand docking techniques have been available in virtual drug screening for specific targets, such as tumor necrosis factor α-converting enzyme (TACE) [18], inducible nitric oxide synthase (iNOS) [19], and Janus-activated kinase 2 (JAK2) [20], these docking approaches to virtual screening are often too computationally expensive [21].

This study aims to investigate the compound-target interaction network of isoquinoline alkaloids of *M. cordata* by reverse pharmacophore database screening technology, and outline its potential action mechanisms.

## Methods

### Workflow

**Fig. 4:**
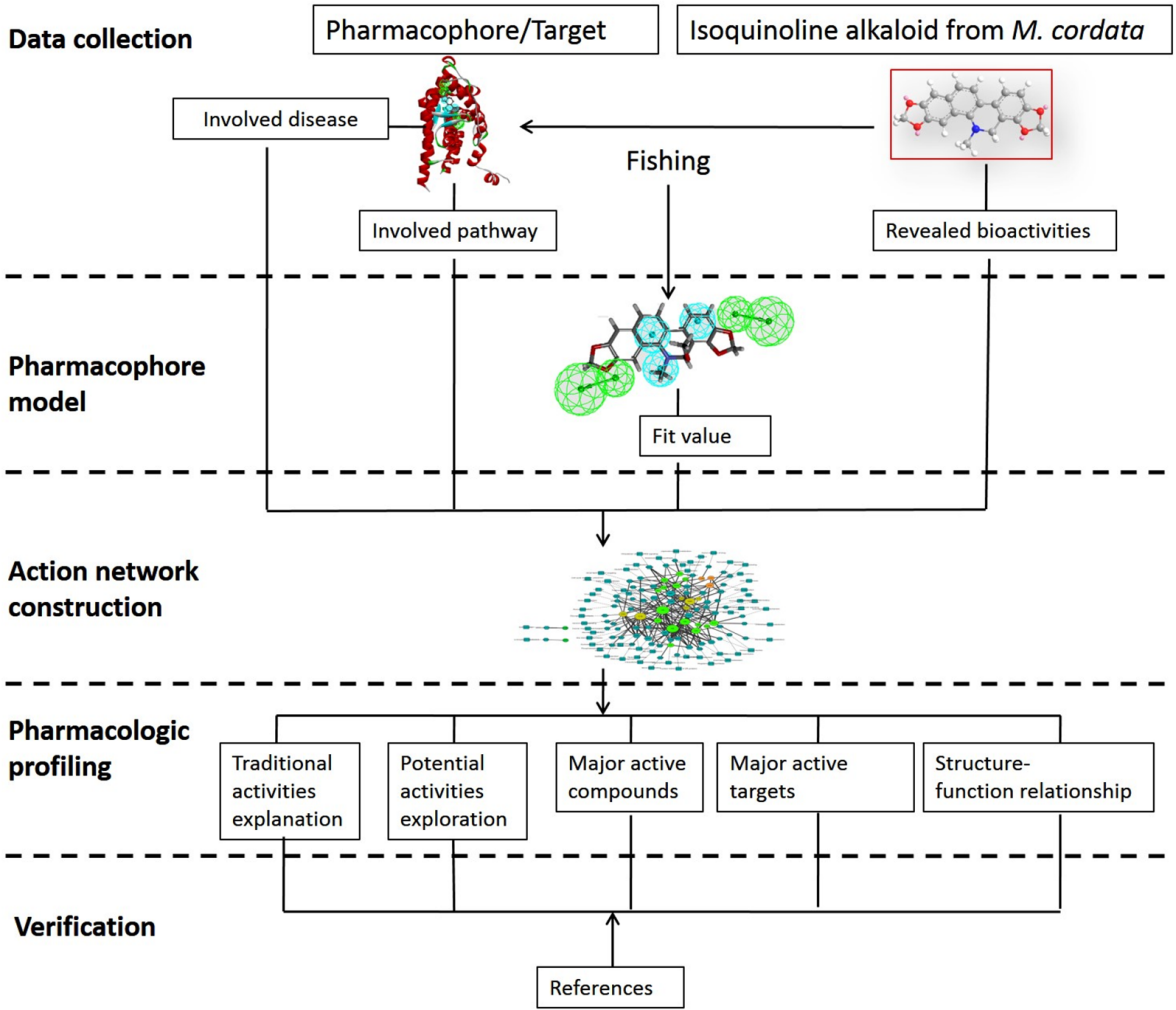
The workflow of this study

Figure 4 shows the workflow of this study. The structures and bioactivities of the isoquinoline alkaloids of *M. cordata* were collected by literature review [17]. The alkaloids were then applied to target fishing with two pharmacophore and target databases, PharmaDB and HypoDB. The hit pharmacophore models were picked out according to the threshold of a predetermined fit value. The results from PharmaDB screening were compared with those from HypoDB screening. After analysis of the hit targets and their associated pathways and diseases, as well as the interactions between the alkaloids and the targets, an action network of *M. cordata* was constructed. Literature retrieval was simultaneously carried out to verify the findings.

### Compound collection

The active components of *M. cordata* were collected from our own database [17] and the literature. All 26 isoquinoline alkaloids of *M. cordata* and their bioactivities are listed in Table 1. As shown in Fig. 2, the alkaloids were divided into three classes: benzo*[c]*phenanthridines (Ben, **C1–C18**), protoberberines (Ber, **C19–C23**), and protopines (Pro, **C24–C26**). Based on the replacement of the C-ring, **C1–C9** belong to the dihydro-benzo*[c]*phenanthridines, **C10** is a *N*-demethyl subtype, and **C11–C16** are quaternary ammonium bases that share an iminium moiety (C=N+). The remaining two bisbenzo*[c]*phenanthridines (BisBen, **C17–C18**) are epimers to one another.

**Table 1:** Basic information of the isoquinoline alkaloids in *M. cordata*

No.	Compounds	Bioactivities	Virtual hitting targets
1	6-Acetonyl-dihydrosanguinarine	Anti-bacteria Insecticidal	MIF; TTR; NQO1
2	6-Acetonyl-dihydrochelerythrine	Anti-oxidant Anti-HIV	HSD1; MIF; PDE4D; NQO1; PLA2s; nAChR 7α; AknH; TtgR
3	6-Methoxy-dihydrochelerythrine	Anti-cancer Anti-parasitic	CAR/RXR; MR; ERα; JNK3; SHBG; AR; 15S-LOX; MMP12; PPARγ; SARS M(pro); Scy D; MAO-A
4	6-Methoxy-dihydrosanguinarine	Anti-bacteria Anti-cancer Anti-platelet aggregation	MR; ERα; FNR; MAO-A
5	Bocconoline	Anti-bacteria Anti-fungal	Opsin 2; HSD1; CAR/RXR; MD; ERα; JNK3; SHBG; Chk1; AR; 15S-LOX; CDK2; CAMKII; Aurora A; PIM1; MMP12; Tankyrase 2; SARS M(pro); PfENR; FabZ; DHODH; CDPKs; FNR; ENR; Scy D; MAO-A
6	Dihydrochelerythrine	Anti-bacteria Anti-fungal	CAR/RXR; MR; ERα; PPO; TTR; JNK3; SHBG; NQO1; RBP4; 15S-LOX; CK2; PIM1; FabZ; DHODH; SnoaL; FNR; ENR; Scy D; MAO-A; MAO-B; AchE; HIV-1 RT; OSBP
7	Dihydrosanguinarine	Anti-bacteria Anti-fungal	MR; ERα; PPO; SHBG; 15S-LOX; CDK2; CK2; MAO-A; AchE
8	Oxysanguinarine	Anti-platelet aggregation	PIM1; CK2
9	Oxychelerythrine	Cytotoxic	CAR/RXR; TTR; JNK3; SHBG; 15S-LOX; CLK1; CK2; PIM1; MMP12; MAPK p38; COMP; FabZ; SonaL; FNR; ENR; MAO-A; MAO-B; AchE; OSBP
10	Norsanguinarine	Anti-fungal	CK2; NmrA
11	6-ethoxychelerythrine	Anti-bacteria Anti-fungal	MIF; TTR; JNK3; GAPDH; nAChR 7α; FabZ; CAT; LmrR; HS5B Pol
12	Chelerythrine	Anti-bacteria Anti-fungal Anti-parasitic Anti-cancer	MIF; TTR; FabZ; HS5B Pol
13	Chelirubine	Anti-proliferative	MIF; NQO1; GR; ZipA-FtsZ; AknH; opdA
14	Macarpine	Cytotoxic Anti-proliferative	PDE4B; PDE 4B; MIF; TTR; NQO1; PIM1; MAPK p38; GR; ZipA-FtsZ; AknH
15	Sanguinarine	Anti-bacteria Anti-fungal Anti-parasitic Anti-cancer Anti-oxidant Hepatotoxicity	MIF; nAChR 7α
16	Sanguilutine	Anti-proliferative	HSD1; MIF; PDE4D; PLA2s; FabZ
17	Sanguidimerine	Unreported	ATTP
18	Chelidimerine	Unreported	MDR HIV-1 Protease
19	Chelanthifoline	Anti-malarial	ALR; ERα; ERβ; MIF; PDK-1; CK2; PIM1; Pi3 Kγ; GR; nAChR 7α; TEM-1; ActR; MAO-B; HIV-1 RT; OSBP
20	Dehydrocicanthifoline	Unreported	HSD1; MR; PDE4B; PDE4D; PPO; MIF; TTR; JNK3; CRBP-2; MAPK p38; AR; PIM1; ZipA-FtsZ; HS5B Pol; HIV-1 RT
21	Berberine	Anti-fungal Anti-malarial Anti-cancer Cytotoxic Anti-inflammatory Anti-Alzheimer’s Anti-fertility Anti-diabetes	MIF; FabZ; Scy D; AchE
22	Dehydrochelanthifoline	Anti-virus	ERα; ERβ; MIF; GSK-3β; TTR; CDK2; PLA2s; MAO-B
23	Coptisine	Cytotoxic Anti-diabetes CYP2D6 inhibition Anti-oxidative Anti-spasmodic	MIF
24	α-Allocryptopine	Anti-funga Anti-arrhythmic	HSD1; MIF; HS5B Pol; Scy D; BACE1
25	β-Allocryptopine	Anti-parasitic Anti-hepatic fibrosis	HSD1; MIF; HS5B POl; Scy D; BACE1; CRALBP; PPO; TTR; nAChR 7α
26	Protopine	Anti-malarial Anti-parasitic Anti-fertility Anti-spasmodic	NQO1; PfENR; TtgR

### Conformation analysis

The structures of all 26 alkaloid candidates were prepared in MOL format, and converted from 2D drawings to 3D models. Their energies were minimized by the software Discovery Studio (DS, v3.5) developed by BIVIA (USA) with the CHARMM force field. A Monte Carlo-based conformational analysis (FAST mode) was performed to generate conformers from the initial conformations. The maximal 255 conformers were allowed with an energy interval of 20 kcal/mol. These alkaloid molecules were rigid, and the number of conformers for each compound was much fewer than 255. Hence, a total of 135 conformers were generated for the 26 isoquinoline alkaloids.

### Ligand profiling

A pharmacophore model represented a series of common features of a set of ligands with a special pharmacological target. The features of a pharmacophore model reflected the target–ligand interaction mode. Pharmacophore-based virtual screening was an alternative to docking. By fitting a compound against a panel of pharmacophore models derived from multiple pharmacological targets, the potential targets of the compound can be outlined.

Automated ligand profiling was available in DS 3.5 as the so-called “Ligand Profiler” protocol. The software offered automated pharmacophore-based activity profiling and reporting [22]. In this study, the default parameters of DS 3.5 were used. For each candidate ligand, three or more features were mapped.

### Pharmacophore databases

DS 3.5 was equipped with two available pharmacophore databases, i.e., HypoDB [23] and PharmaDB [24]. HypoDB contained about 2500 pharmacophore models derived from protein–ligand 3D complex structures as well as structural data on small bioactive organic molecules. PharmaDB was created from the sc-PDB, a well-accepted data source in structure-based profiling protocols. The sc-PDB was a collection of 3D structures of binding sites found in the Protein Data Bank (PDB). The binding sites were extracted from crystal structures in which a complex between a protein cavity and a small molecule ligand could be identified. PharmaDB consisted of about 68,000 pharmacophores derived from 8000 protein–ligand complexes from the sc-PDB dataset. PharmaDB is a new and updated pharmacophore database developed in collaboration with Prof. Didier Rognan [25, 26]. The target and pharmacophore models from PharmaDB and HypoDB were not entirely consistent. PharmaDB had a larger quantity of targets, while the models in the HypoDB were fewer and described as being experimentally validated. Therefore, in this study, PharmaDB was employed in the target fishing, and HypoDB was used to validate the results.

Regarding PharmaDB, multiple pharmacophores with shape or excluded volume constraints were generated for each protein target. For the pharmacophores with shape constraints, the suffix “-s” was added to the name. In addition, a numerical suffix referred to the ranking of selectivity evaluated by a default algorithm in DS v3.5. In this study, only the best models with “−1” in their names were employed in the ligand profiling [23]. For each pharmacophore database, a classification tree was available, from which the individual models could be selected.

### Parameters

In the profiling with PharmaDB, all the pharmacophore models with the shape of the binding pocket were selected for the virtual screening with default settings. The RIGID mode was used as the molecular mapping algorithm. No molecular features were allowed to be missed while mapping these ligands to the pharmacophore models to increase selectivity. The minimal inter-feature distance was set at 0.5 Å. Parallel screening technology for one or more compounds against a multitude of pharmacophore models was available as a Pipeline Pilot protocol. The number of parallel processing procedures was set at 4. The whole calculation was carried on a T5500 workstation (DELL inc., USA).

### Binding mode refinement

All the poses of the ligands mapped into the pharmacophore were preserved. A series of target-ligand pairs were selected as emphasis for further examinations. The selection was based upon compatibility with the reported pharmacological activities, as well as traditional usage of *M.cordata*. A further refinement was carried out in Molecular Operating Environment (MOE) developed by CCG (Canada) to identify the protein–ligand binding modes. Energy minimization was carried out by conjugated gradient minimization with the MMFF94x force field, until an RMSD of 0.1 kcal mol^−1^ Ǻ^−1^ was reached.

### Network construction

An interaction table between alkaloids and targets was presented as the ligand profiling results. For each target, the name and pathway information were collected from the PDB and KEGG. The diseases related to the targets were collected from the Therapeutic Target Database (TTD; http://bidd.nus.edu.sg/group/cjttd/) [27] and DrugBank (http://www.drugbank.ca/) [28] databases. Compound-Target-Pathway networks were generated by Cytoscape 3.0 (Cytoscape Consortium, USA) [29]. In the networks, nodes represented the compounds, targets, and biological pathways. The edges linking the compound-target and target-pathway represented their relationships and were marked with different types of lines. After the network was built, the basic parameters of the network were computed and analyzed.

## Results and discussion

The profiling results are presented in two HTML tables, designated MoleculeFits and PharmacophoreFits. Two descriptors, fit value and shape similarity, were used to measure the fitness of the ligand and pharmacophore. A fit value equal to or greater than 0.3 was used as a heuristic threshold to select targets from the activity profiler. For each pharmacophore model, the classification information of the target can be indicated in a HTML table created by DS 3.5 called as Pharmacophores. Finally, 98 pharmacophore models were mapped. The models belonged to 65 protein targets, and were involved in 60 pathways. A complete list of the 241 target-ligand pairs is shown in Table 2. The name and indication information of the targets are shown in Table 3. The 13 targets verified by HypoDB screening are marked with an asterisk in Table 3.

**Table 2:** The results of ligand profiling

Class	CMD-ID	ph4	Target short name	Gene	Uniprot-AC	Fit value	Shape similarity
Ben	1	3cfn	TTR	TTHY_HUMAN	P02766	0.750635	0.508475
Ben	1	1h69	NQO1	NQO1_HUMAN	P15559	0.923086	0.536437
Ben	2	3kba	Progesterone receptor	PRGR_HUMAN	P06401	0.334698	0.506897
Ben	2	1xom	PDE4D	PDE4D_HUMAN	Q08499	0.346437	0.527574
Ben	2	2wnj	nAChR 7α	Q8WSF8_APLCA	Q8WSF8	0.43985	0.505495
Ben	2	1h69	NQO1	NQO1_HUMAN	P15559	0.928518	0.504604
Ben	3	2oz7	AR	ANDR_HUMAN	P10275	0.360685	0.500849
Ben	3	2a3i	MR	MCR_HUMAN	P08235	0.375601	0.528195
Ben	3	5std	ScyD	SCYD_MAGGR	P56221	0.418672	0.543119
Ben	3	1l2i	ERα	ESR1_HUMAN	P03372	0.420147	0.542969
Ben	3	1xvp	CAR/RXR	NR1I3_HUMAN	Q14994	0.460385	0.534672
Ben	3	3lmp	PPARγ	PPARG_HUMAN	P37231	0.526039	0.500787
Ben	3	1d2s	SHBG	SHBG_HUMAN	P04278	0.558685	0.563525
Ben	3	2gz7	SARS M(pro)	R1AB_CVHSA	P0C6X7	0.559512	0.547348
Ben	3	2p0m	15S-LOX	LOX15_RABIT	P12530	0.639897	0.537344
Ben	3	3f15	MMP12	MMP12_HUMAN	P39900	0.725254	0.50503
Ben	3	2bxr	MAO-A	AOFA_HUMAN	P21397	0.799156	0.521008
Ben	3	2o2u	JNK3	MK10_HUMAN	P53779	0.835637	0.577825
Ben	4	2bgi	FNR	Q9L6V3_RHOCA	Q9L6V3	0.427793	0.516878
Ben	4	1l2i	ERα	ESR1_HUMAN	P03372	0.452535	0.593291
Ben	4	2bxr	MAO-A	AOFA_HUMAN	P21397	0.793632	0.533917
Ben	5	2ol4	PfENR	Q9BH77_PLAFA	Q9BH77	0.315455	0.518182
Ben	5	3g0u	DHODH	PYRD_HUMAN	Q02127	0.369491	0.508604
Ben	5	2bxr	MAO-A	AOFA_HUMAN	P21397	0.387312	0.544118
Ben	5	2a3i	MR	MCR_HUMAN	P08235	0.387489	0.563771
Ben	5	7std	ScyD	SCYD_MAGGR	P56221	0.422146	0.504744
Ben	5	2uue	CDK2	CDK2_HUMAN	P24941	0.424268	0.567108
Ben	5	2oz7	AR	ANDR_HUMAN	P10275	0.426337	0.510961
Ben	5	3coh	Aurora-A	STK6_HUMAN	O14965	0.4511	0.531532
Ben	5	3kr8	Tankyrase 2	TNKS2_HUMAN	Q9H2K2	0.464801	0.548729
Ben	5	1d2s	SHBG	SHBG_HUMAN	P04278	0.480784	0.571721
Ben	5	1l2i	ERα	ESR1_HUMAN	P03372	0.493882	0.57529
Ben	5	2wel	CAMKII	KCC2D_HUMAN	Q13557	0.493929	0.516729
Ben	5	3fne	ENR	INHA_MYCTU	P0A5Y6	0.50498	0.546169
Ben	5	1xvp	CAR/RXR	NR1I3_HUMAN	Q14994	0.508683	0.576427
Ben	5	5std	ScyD	SCYD_MAGGR	P56221	0.522671	0.566972
Ben	5	2brg	Chk1	CHK1_HUMAN	O14757	0.541427	0.51711
Ben	5	3doz	FabZ	Q5G940_HELPY	Q5G940	0.546379	0.507843
Ben	5	2bgi	FNR	Q9L6V3_RHOCA	Q9L6V3	0.553334	0.549296
Ben	5	3fnf	ENR	INHA_MYCTU	P0A5Y6	0.562321	0.511494
Ben	5	3fnh	ENR	INHA_MYCTU	P0A5Y6	0.614823	0.507547
Ben	5	2p0m	15S-LOX	LOX15_RABIT	P12530	0.673148	0.541414
Ben	5	3dp1	FabZ	Q5G940_HELPY	Q5G940	0.687924	0.53816
Ben	5	2gz7	SARS M(pro)	R1AB_CVHSA	P0C6X7	0.694125	0.551789
Ben	5	2o2u	JNK3	MK10_HUMAN	P53779	0.805153	0.553719
Ben	5	3f15	MMP12	MMP12_HUMAN	P39900	0.893862	0.507187
Ben	6	3fj6	DHODH	PYRD_HUMAN	Q02127	0.341464	0.566038
Ben	6	5std	ScyD	SCYD_MAGGR	P56221	0.364451	0.555556
Ben	6	1d2s	SHBG	SHBG_HUMAN	P04278	0.367805	0.529289
Ben	6	2a3i	MR	MCR_HUMAN	P08235	0.368001	0.559289
Ben	6	1xvp	CAR/RXR	NR1I3_HUMAN	Q14994	0.423023	0.572534
Ben	6	2v60	MAO-B	AOFB_HUMAN	P27338	0.436774	0.511294
Ben	6	1l2i	ERα	ESR1_HUMAN	P03372	0.451108	0.529175
Ben	6	1rbp	RBP4	RET4_HUMAN	P02753	0.486949	0.516378
Ben	6	2nsd	ENR	INHA_MYCTU	P0A5Y6	0.509088	0.530738
Ben	6	1kgj	TTR	TTHY_RAT	P02767	0.522255	0.529412
Ben	6	1tv6	HIV-1 TR	POL_HV1B1	P03366	0.564494	0.529981
Ben	6	2bgi	FNR	Q9L6V3_RHOCA	Q9L6V3	0.636419	0.536325
Ben	6	2p0m	15S-LOX	LOX15_RABIT	P12530	0.663082	0.545045
Ben	6	3imu	TTR	TTHY_HUMAN	P02766	0.690803	0.577011
Ben	6	3dp1	FabZ	Q5G940_HELPY	Q5G940	0.705916	0.565401
Ben	6	2o2u	JNK3	MK10_HUMAN	P53779	0.705945	0.507463
Ben	6	1h69	NQO1	NQO1_HUMAN	P15559	0.755827	0.508911
Ben	6	2bxr	MAO-A	AOFA_HUMAN	P21397	0.795152	0.541573
Ben	6	1sjw	SnoaL	Q9RN59_STRNO	Q9RN59	0.904111	0.661572
Ben	6	2j3q	AChE	ACES_TORCA	P04058	0.992134	0.661327
Ben	7	2x1n	CDK2	CDK2_HUMAN	P24941	0.329358	0.521253
Ben	7	1d2s	SHBG	SHBG_HUMAN	P04278	0.340019	0.542857
Ben	7	1l2i	ERα	ESR1_HUMAN	P03372	0.465563	0.553846
Ben	7	2j3q	AChE	ACES_TORCA	P04058	0.470546	0.67
Ben	7	2p0m	15S-LOX	LOX15_RABIT	P12530	0.639874	0.545254
Ben	7	2bxr	MAO-A	AOFA_HUMAN	P21397	0.822509	0.548694
Ben	8	3bgp	PIM-1	PIM1_HUMAN	P11309	0.659102	0.52193
Ben	9	2wu7	CLK1	CLK3_HUMAN	P49761	0.333353	0.541053
Ben	9	1d2s	SHBG	SHBG_HUMAN	P04278	0.40988	0.526096
Ben	9	2nsd	ENR	INHA_MYCTU	P0A5Y6	0.417703	0.522727
Ben	9	1tha	TTR	TTHY_HUMAN	P02766	0.42188	0.505071
Ben	9	1xvp	CAR/RXR	NR1I3_HUMAN	Q14994	0.459386	0.600775
Ben	9	1fbm	COMP	COMP_RAT	P35444	0.540756	0.509542
Ben	9	2p0m	15S-LOX	LOX15_RABIT	P12530	0.609094	0.548596
Ben	9	2bxr	MAO-A	AOFA_HUMAN	P21397	0.636415	0.524336
Ben	9	3iw7	MAPK p38	MK14_HUMAN	Q16539	0.671331	0.532803
Ben	9	1sjw	SnoaL	Q9RN59_STRNO	Q9RN59	0.679871	0.665953
Ben	9	2bgi	FNR	Q9L6V3_RHOCA	Q9L6V3	0.68446	0.509554
Ben	9	3dp1	FabZ	Q5G940_HELPY	Q5G940	0.723083	0.601732
Ben	9	2v60	MAO-B	AOFB_HUMAN	P27338	0.818911	0.501006
Ben	9	2o2u	JNK3	MK10_HUMAN	P53779	0.878824	0.532609
Ben	9	2j3q	AChE	ACES_TORCA	P04058	0.992667	0.679157
Ben	10	2wmd	NmrA	NMRL1_HUMAN	Q9HBL8	0.635677	0.601671
Ben	11	3doz	FabZ	Q5G940_HELPY	Q5G940	0.380298	0.514677
Ben	11	3kvx	JNK3	MK10_HUMAN	P53779	0.408174	0.518987
Ben	11	2wnj	nAChR 7α	Q8WSF8_APLCA	Q8WSF8	0.408648	0.512476
Ben	11	3doy	FabZ	Q5G940_HELPY	Q5G940	0.456816	0.515444
Ben	11	1k3t	GAPDH	G3PG_TRYCR	P22513	0.56209	0.500931
Ben	11	3lmp	PPARγ	PPARG_HUMAN	P37231	0.648243	0.508527
Ben	11	1qca	CAT	CAT3_ECOLX	P00484	0.780585	0.505747
Ben	11	3f8f	LmrR	A2RI36_LACLM	A2RI36	0.817481	0.51932
Ben	13	1xan	GR	GSHR_HUMAN	P00390	0.573697	0.520833
Ben	13	3kba	Progesterone receptor	PRGR_HUMAN	P06401	0.651629	0.522059
Ben	13	1h69	NQO1	NQO1_HUMAN	P15559	0.811055	0.503055
Ben	13	3a3w	opdA	Q93LD7_RHIRD	Q93LD7	0.833715	0.510158
Ben	14	3huc	MAPK p38	MK14_HUMAN	Q16539	0.345074	0.534091
Ben	14	1xan	GR	GSHR_HUMAN	P00390	0.517848	0.510823
Ben	14	1h69	NQO1	NQO1_HUMAN	P15559	0.723867	0.515504
Ben	14	1xom	PDE4D	PDE4D_HUMAN	Q08499	0.745933	0.503704
Ben	14	1xlx	PDE4B	PDE4B_HUMAN	Q07343	0.796555	0.52037
Ben	15	2wnj	nAChR 7α	Q8WSF8_APLCA	Q8WSF8	0.495424	0.509356
Ben	16	3kba	Progesterone receptor	PRGR_HUMAN	P06401	0.342606	0.537671
Ben	16	1xom	PDE4D	PDE4D_HUMAN	Q08499	0.816692	0.539427
BisBen	17	1r5 l	ATTP	TTPA_HUMAN	P49638	0.32356	0.514156
BisBen	18	1rq9	MDR HIV-1 Protease	Q5RTL1_9HIV	Q5RTL1	0.621229	0.507743
Ber	19	1u3s	ERβ	ESR2_HUMAN	Q92731	0.408794	0.535377
Ber	19	2j3q	AChE	ACES_TORCA	P04058	0.485705	0.596737
Ber	19	3l54	Pi3 Kγ	PK3CG_HUMAN	P48736	0.498919	0.59589
Ber	19	1pzo	TEM-1	BLAT_ECOLX	P62593	0.523404	0.526667
Ber	19	2ikg	ALR	ALDR_HUMAN	P15121	0.561704	0.507109
Ber	19	1c1c	HIV-1 TR	POL_HV1H2	P04585	0.577074	0.542373
Ber	19	2r7b	PDK-1	PDPK1_HUMAN	O15530	0.587223	0.533049
Ber	19	1yye	ERβ	ESR2_HUMAN	Q92731	0.679767	0.56691
Ber	19	1qkt	ERα	ESR1_HUMAN	P03372	0.681913	0.56351
Ber	19	1xan	GR	GSHR_HUMAN	P00390	0.715506	0.548544
Ber	19	2wnj	nAChR 7α	Q8WSF8_APLCA	Q8WSF8	0.87937	0.501031
Ber	19	3b6c	ActR	Q53901_STRCO	Q53901	0.880577	0.597561
Ber	19	1x78	ERβ	ESR2_HUMAN	Q92731	0.909059	0.522565
Ber	20	1xm4	PDE4B	PDE4B_HUMAN	Q07343	0.402388	0.569138
Ber	20	1tha	TTR	TTHY_HUMAN	P02766	0.45615	0.514286
Ber	20	1tv6	HIV-1 TR	POL_HV1B1	P03366	0.459002	0.521154
Ber	20	2nw4	AR	ANDR_RAT	P15207	0.463505	0.541203
Ber	20	1opb	CRBP2	RET2_RAT	P06768	0.485837	0.534653
Ber	20	2waj	JNK3	MK10_HUMAN	P53779	0.572085	0.603104
Ber	20	1kgj	TTR	TTHY_RAT	P02767	0.740087	0.56531
Ber	20	1xom	PDE4D	PDE4D_HUMAN	Q08499	0.811727	0.542406
Ber	20	1xlx	PDE4B	PDE4B_HUMAN	Q07343	0.859016	0.51341
Ber	20	3i6d	PPO	PPOX_BACSU	P32397	0.97618	0.570499
Ber	21	5std	ScyD	SCYD_MAGGR	P56221	0.324086	0.505682
Ber	21	2j3q	AChE	ACES_TORCA	P04058	0.992907	0.672209
Ber	22	1di8	CDK2	CDK2_HUMAN	P24941	0.406566	0.501094
Ber	22	1u3s	ERβ	ESR2_HUMAN	Q92731	0.661777	0.509434
Pro	24	5std	ScyD	SCYD_MAGGR	P56221	0.538905	0.543636
Pro	24	3ine	BACE1	BACE1_HUMAN	P56817	0.54561	0.522968
Pro	25	1tyr	TTR	TTHY_HUMAN	P02766	0.356425	0.526412
Pro	25	3inf	BACE1	BACE1_HUMAN	P56817	0.37118	0.504303
Pro	25	2wnj	nAChR 7α	Q8WSF8_APLCA	Q8WSF8	0.553029	0.533461
Pro	25	3ine	BACE1	BACE1_HUMAN	P56817	0.597712	0.51259
Pro	25	3hx3	CRALBP	RLBP1_HUMAN	P12271	0.604625	0.513158
Pro	25	5std	ScyD	SCYD_MAGGR	P56221	0.763034	0.522523
Pro	26	2ow2	PfENR	MMP9_HUMAN	P14780	0.302479	0.507865
Pro	26	2f1o	NQO1	NQO1_HUMAN	P15559	0.432407	0.52183

**Table 3:** The targets identified

Targets	Short name	Type	Pathway	Diseases
Retinaldehyde-binding protein	CRALBP	Research	Retinaldehyde metabolism	Retinitis pigmentosa
Rhodopsin	Opsin 2	Research	Retina metabolism	Retinitis pigmentosa
11-Beta-hydroxysteroid dehydrogenase	HSD1	Successful	Glucocorticoid concentration	Diabetes Osteoporosis Hepatotoxicity
CAR/RXR heterodimer	CAR/RXR	Research	Triglyceride metabolism	Diabetes Hepatitis
Aldose reductase	ALR	Successful	Glucolipid metabolism	Diabetes Pain
Mineralocorticoid receptors	MR	Successful	Na^+^/K^+^ equilibrium	Inflammatory, autoimmune disease Injury
Phosphodiesterase 4B	PDE4B	Successful	AKT/mTOR pathway	Cancer Obesity
Phosphodiesterase 4D	PDE4D	Successful	Intracellular cAMP//CREB signaling	Cancer Alzheimer’s
Protoporphyrinogen oxidase	PPO	Research	Heme biosynthesis	Cancer Parasitosis
Transthyretin	TTR	Clinic Trial	Thyroxine carrier	Cancer Alzheimer’s
Mitogen-activated protein kinase 10	JNK3	Research	GbRH/ErbB/MAPK/insulin signaling pathway	Cancer Alzheimer’s
Sex hormone-binding globulin	SHBG	Research	Sex steroids biosynthesis	Cancer
NAD(P)H:quinone oxidoreductase	NQO1	Research	Quinones metabolism	Cancer
Cellular retinol binding protein II	CRBP2	Research	Retinol metabolism	Cancer
Estrogen receptor alpha^a^	ERα^a^	Successful	Estrogen metabolism Insulin-like growth factor pathway	Cancer Alzheimer’s Injury Osteoporosis
Alpha-tocopherol (alpha-T) transfer protein	ATTP	Research	α-Tocopherol metabolism	Cancer
Human serum retinol binding protein 4	RBP4	Research	Retinol metabolism	Cancer
Estrogen receptor beta^a^	ERβ^a^	Successful	Estrogen metabolism MAPK, PI3K signaling	Cancer Alzheimer’s Injury
Checkpoint kinase 1^a^	Chk1^a^	Research	DNA damage response	Cancer
Androgen receptor	AR	Successful	Hormone metabolism	Cancer
Reticulocyte 15S-lipoxygenase	15S-LOX	Research	Arachidonic acid metabolism	Cancer
3-Phosphoinositide-dependent kinase-1^a^	PDK-1^a^	Research	Phosphatidylinositol 3 kinase (PI3K) signaling	Cancer
Casein kinase 2^a^	CK2^a^	Research	Ser/Thr pathway	Cancer
Cyclin dependent kinase 2^a^	CDK2^a^	Research	Cell cycle	Cancer
Calcium/calmodulin dependent protein kinase II delta	CAMKII	Research	NF-κB-mediated inflammatory response Ca^2+^-linked signaling	Cancer Inflammatory, autoimmune disease
Dual-specificity protein kinase 1	CLK1	Research	Nuclear redistribution of SR proteins	Cancer
Proto-oncogene serine threonine kinase^a^	PIM-1^a^	Research	Cell cycle regulation JAK/STAT pathway	Cancer
Aurora kinase A	Aurora-A	Clinical trial	Cell cycle arrest	Cancer
Matrix metalloproteinases	MMP12	Research	Cell invasion, metastasis	Cancer Inflammatory, autoimmune disease
Phospholipase A2	PLA2s	Successful	VEGF/MAPK/GnRH signaling	Cancer Inflammatory, autoimmune disease
Mitogen-Activated Protein Kinases p38	MAPK p38	Clinical trial	MAPK signaling	Cancer Pain Inflammatory, autoimmune disease Dermatosis
Tankyrase 2	Tankyrase 2	Research	Canonical Wnt signaling	Cancer
Phosphatidylinositol-4,5-bisphosphate 3-kinase catalytic subunit gamma isoform	Pi3Kγ	Research	Cancer migration, invasion Inositol phosphate metabolism	Cancer Inflammatory, autoimmune disease
PPARgamma-LBD^a^	PPARγ^a^	Research	LPS-induced iNOS expression	Cancer Inflammatory, autoimmune disease Osteoporosis
Cartilage oligomeric matrix protein	COMP	Research	Bone regeneration	Autoimmune disease Injury
Severe acute respiratory syndrome coronavirus (SARS-CoV) main protease (M(pro))	SARS M(pro)	Research	Virus maturation	Virus infection
Glycosomal glyceraldehyde-3-Phosphate Dehydrogenase	GAPDH	Successful	Glyceraldehydes metabolism	Parasitosis
Glutathione disulfide oxidoreductase	GR	Research	Glutathione metabolism	Parasitosis
Acyl carrier protein reductase^a^	PfENR^a^	Successful	Fatty acid biosynthesis	Parasitosis
Acetylcholine binding protein alpha7	nAChR 7α	Successful	Calcium signaling pathway	Alzheimer’s Pain
3R-hydroxyacyl-acyl carrier protein dehydratase	FabZ	Research	Fatty acid biosynthesis	Parasitosis
Dihydroorotate dehydrogenase	DHODH	Successful	Pyrimidine metabolism	Parasitosis
TEM-1 Beta-Lactamase^a^	TEM-1^a^	Successful	Cefotaxime metabolism	Bacterial infection
Chloramphenicol acetyltransferase	CAT	Research	Chloramphenicol metabolism	Bacterial infection
Polyketide cyclase SnoaL	SnoaL	Research	Nogalamycin biosynthesis	Bacterial infection
ZipA attaches FtsZ protein	ZipA-FtsZ	Research	Cell division	Bacterial infection
Ferredoxin-NADP^+^ reductase	FNR	Successful	Redox metabolism	Bacterial infection
Polyketide cyclase AknH	AknH	Research	Aclacinomycin biosynthesis	Bacterial infection
Enoyl-acyl carrier protein reductase	ENR	Successful	Fatty acid biosynthesis	Bacterial infection
Multidrug binding protein TtgR	TtgR	Research	Active extrusion of drug	Bacterial infection
NmrA-like family domain	NmrA	Research	Transcriptional repress	Fungal infection
Bacterial phosphotriesterase	opdA	Research	Organophosphate metabolism	Bacterial infection
Streptomyces coelicolor TetR family protein ActR^a^	ActR^a^	Research	Transcriptional repress	Bacterial infection
Multidrug binding transcriptional regulator LmrR	LmrR	Research	Autoregulatory mechanism	Bacterial infection
Scytalone Dehydratase	ScyD	Research	Fungicide	Fungal infection
Human monoamine oxidase A	MAO-A	Successful	Monoamines metabolism	Depression
Acetylcholin esterase	AChE	Successful	Glycerophospholipid metabolism	Alzheimer’s Parkinson’s
β-Site amyloid precursor protein cleaving enzyme	BACE1	Clinical trial	Neuregulin processing	Alzheimer’s
Multidrug-resistant HIV-1 protease^a^	MDR HIV-1 protease^a^	Successful	Self-activation	AIDs
HIV-1 reverse transcriptase	HIV-1 TR	Successful	ATP-dependent excision, pyrophosphorolysis	AIDs
Oxysterol binding protein	OSBP	Research	Intracellular lipid homeostasis Signal conduction	Virus infection Cancer
Rhodopsin	Opsin 2	Research	Rod photoreceptor	Retinitis pigmentosa
Macrophage migration inhibitory factor	MIF	Clinical trial	Phenylalanine, tyrosine metabolism	Cancer Inflammatory, autoimmune disease
Glycogen synthase kinase-3 beta	GSK-3β	Research	Glycogen biosynthesis	Cancer Alzheimer’s Diabetes
Hepatitis C virus (HCV) polymerase	HS5B Pol	Successful	DNA biosynthesis	Virus infection

^a^The targets verified by HypoDB screening

### Analysis of the interaction network

A topological analysis of the interaction network offered insights into the biologically relevant connectivity patterns, and highly influential compounds or targets. Some Chinese medicines had been investigated by interaction network analysis [30–32].

**Fig. 5:**
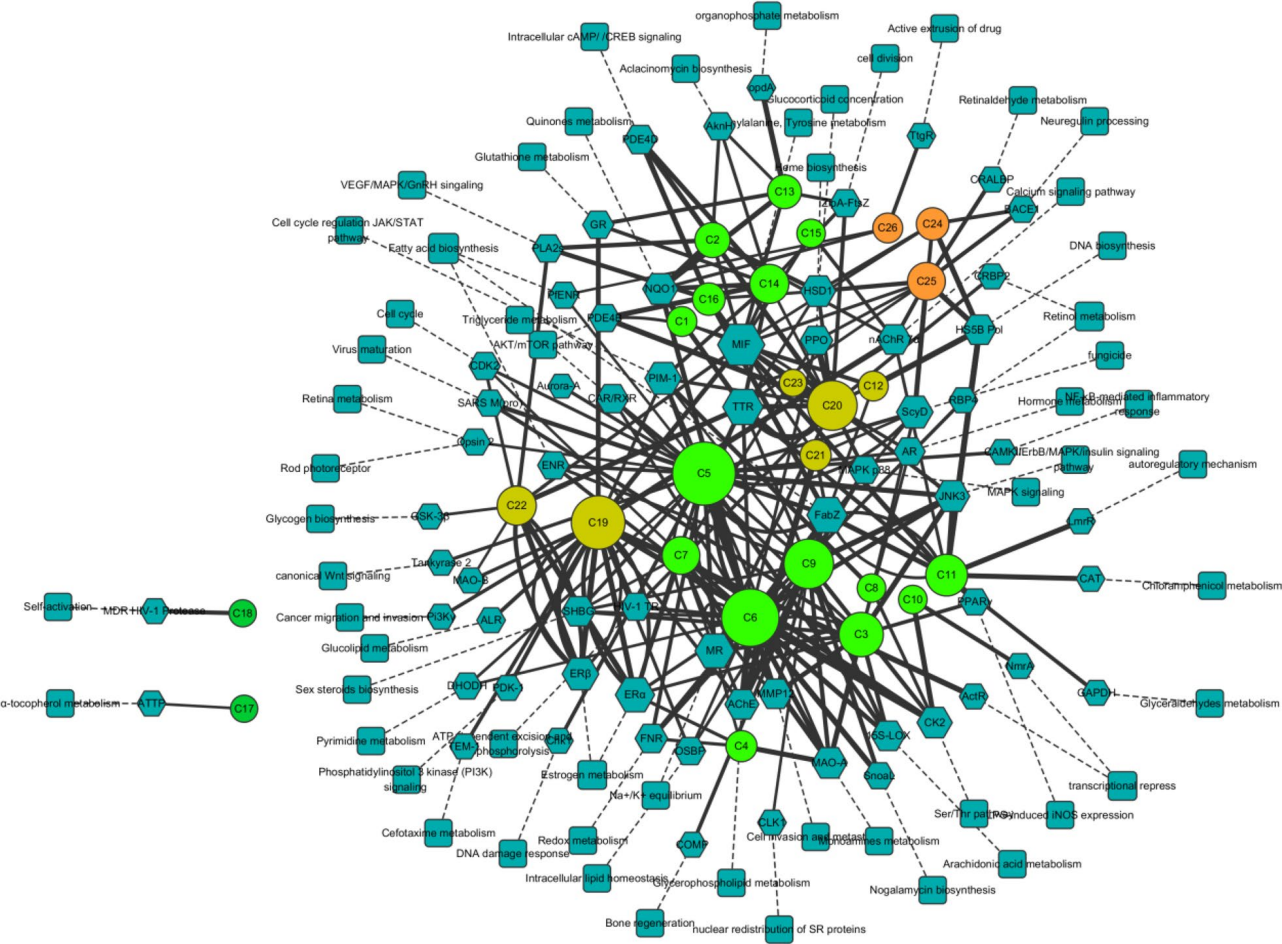
The pharmacological network of *Macleaya cordata*. Hexagon, targets; Rectangle, biopathway; Ellipse, alkaloids (*bright green* Ben, *dark green* BisBen, *breen* Ber, *orange* Pro)

The pharmacological network of *M. cordata* had three types of nodes (Fig. 5). The 26 alkaloid nodes formed the core of the network, and were surrounded by 65 target nodes. Each target was linked to at least one pathway. A total of 60 pathway nodes constituted the outer layer of the network. Each alkaloid was the center of a star-shaped action net except for the two bisbenzo*[c]*phenanthridines (BisBen), which were only linked to one target and one pathway, respectively. The alkaloids and targets were strongly interconnected in many-to-many relationships.

**Fig. 6:**
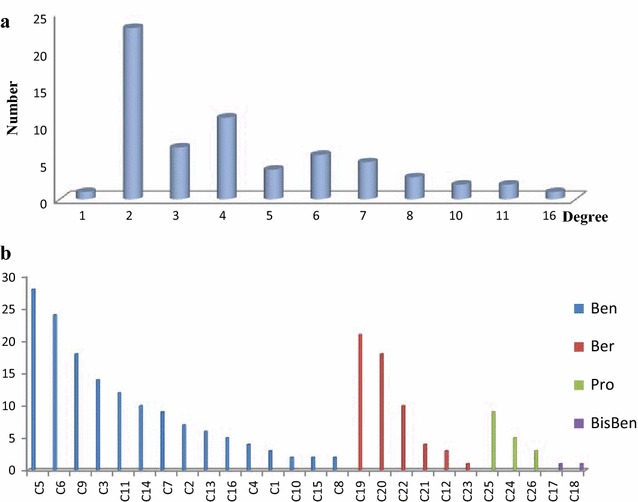
Degree distribution in the network. **a** alkaloids, **b** targets

A general overview of the global topological properties of the network was obtained from the statistical data by the Network Analyzer of Cytoscape. The diameter of the network was 8.0, the centralization was 0.14, and the density was 0.024. The node degree indicated the number of edges linking to other nodes. The highly connected nodes were referred to as the hubs of the network. The degrees of all the alkaloids (Fig. 6a) and important targets (Fig. 6b) were investigated. The compounds with higher degree values, such as **C5**, **C6**, **C9**, **C19**, and **C20**, that might participate in more interactions than the other components were the hubs in the network. The target degree values mostly ranged between 2 and 7. The targets with the highest degree values included MIF (16), TTR (11), FabZ* (11), ERα* (10), and MR (10). The targets with higher degree values might be involved in the pharmacological actions of *M. cordata.*

### Interpreting the pharmacological actions

By mining the PubMed and TTD, the targets of *M. cordata* in the PharmaDB profiling results were annotated with biological functions and clinical indications (Table 3). Furthermore, the targets were classified according to the reported pharmacological activities of *M. cordata* as follows: microorganism (including bacterial, fungal, and viral) infection (12 targets, with 3 targets verified by HypoDB screening), parasitic disease (5 targets, with 2 targets validated by HypoDB screening), pain (3 targets), cancer (31 targets, with 8 targets confirmed by HypoDB screening), inflammation (8 targets, with 1 target verified by HypoDB screening), and injury (4 targets, with 2 targets fished by HypoDB screening).

#### Antibacterial activity

**Fig. 7:**
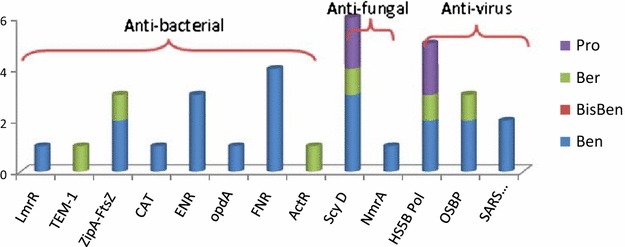
The compounds mapping of microorganism related targets

The extracts and their purified alkaloids from *M. cordata* exhibited notable activities against *Staphylococcus aureus*, *Pseudomonas aeruginosa*, *Escherichia coli*, *Bacillus subtilis*, *Tetracoccus* spp., and methicillin-resistant *Staphylococcus aureus* (MRSA) [12, 33]. In this study, 12 proposed targets were closely related to microorganisms, and seven of them exhibited antibacterial activities (Fig. 7). the key types of alkaloids with antibacterial activity were dihydro-benzo*[c]*phenanthridine alkaloids and protoberberines.

Five targets (LmrR, TEM-1*, CAT, FNR, and ActR) were related to multidrug-resistant bacterial strains. LmrR, a multidrug binding transcriptional regulator and the predicted target of **C11**, was a PadR-related transcriptional repressor that regulated the production of LmrCD, a major multidrug ABC transporter in *Lactococcus lactis* [34, 35]. TEM-1* (TEM-1 *beta*-lactamase) fished by **C19** was one of the antibiotic-resistance determinants for penicillins, early cephalosporins, and novel drugs from their derivatives [36]. A new drug, Avibactam™, innovated by AstraZeneca is a TEM-1 inhibitor that has already entered phase III clinical development [37]. In addition, chloramphenicol acetyltransferase (CAT), an antibiotic-inactivating enzyme predicted by **C11**, catalyzed the acetyl-*S*-CoA-dependent acetylation of chloramphenicol at the 3-hydroxyl group and resulted in chloramphenicol-resistance in bacteria [38]. Ferredoxin-NADP^+^ reductase (FNR), targeted in silico by **C4**, **C5**, **C6**, and **C9**, participated in numerous electron transfer reactions, had no homologous enzyme in humans, and was a target for the accumulation of multidrug-resistant microbial strains [39]. The *Streptomyces coelicolor* TetR family protein ActR* was found by **C19**. ActR* may mediate timely self-resistance to an endogenously-produced antibiotic. TetR-mediated antibiotic-resistance might have been acquired from an antibiotic-producer organism [40].

**Fig. 8:**
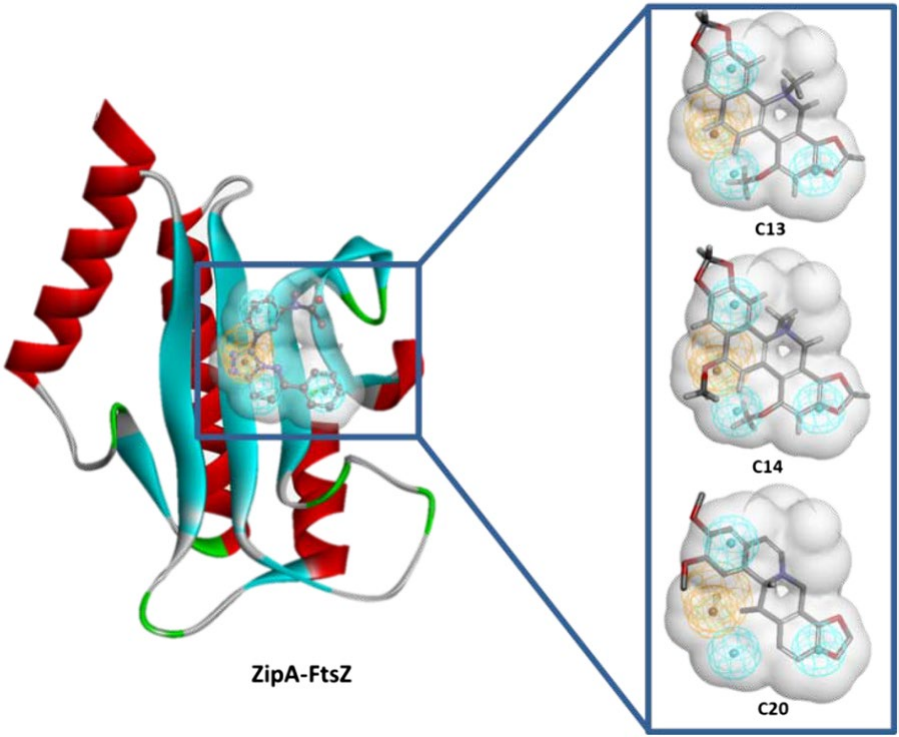
Three alkaloids mapped to ZipA-FtsZ. *Left* the crystal structure and pharmacophore of target, *right* the alkaloids fit to the pharmacophore

Two targets indicating other pathways were involved in the antibacterial activity. The ZipA-FtsZ complex was fished by **C13**, **C14**, and **C20** (Fig. 8). ZipA was a membrane-anchored protein in *E. coli* that interacted with FtsZ-mediated bacterial cell division, and was considered a potential target for antibacterial agents [41]. The target ENR catalyzed an essential step in fatty acid biosynthesis. ENR was a target for narrow-spectrum antibacterial drug discovery because of its essential role in metabolism and its sequence conservation across many bacterial species [42].

#### Antiparasitic activity

*M. cordata* showed remarkable effects against *Ichthyophthirius multifiliis* in grass carp [43] and richadsin [44], as well as against *Dactylogyrus intermedius* in *Carassius auratus* [45]. The total alkaloids of *M. cordata* were able to kill gastrointestinal parasites [46].

In this study, five targets involved in parasitic diseases were predicted. Because of the lack of reported protein–ligand crystal structures for parasitosis, these five targets were not related to the above parasitosis in either humans or other animals. However, the findings suggested the potential of *M.cordata* to treat other parasitosis, such as malaria, Chagas disease, and Kala-azar. The enoyl-acyl carrier reductase PfENR* fished by two alkaloids (**C5** and **C26**) and the (3R)-hydroxymyristoyl acyl carrier protein dehydratase FabZ* in silico targeted by six alkaloids (**C5**, **C6**, **C9**, **C11**, **C12**, and **C16**) were involved in the fatty acid biosynthesis of *Plasmodium falciparum*. The antioxidant enzyme GR fished by **C13**, **C14**, and **C19** was a target for antimalarial drug development [47]. The target glycosomal glyceraldehyde-3-phosphate dehydrogenase (GAPDH) found by **C11** was a target for the development of novel chemotherapeutic agents for the treatment of Chagas disease [48]. Dihydroorotate dehydrogenase (DHODH) retrieved by **C5** and **C6** was related to both *Leishmania* infection and *Trypanosoma* infection [49].

#### Analgesic activity

A mixture of the isoquinoline alkaloids from *M. cordata* exhibited strong analgesic activity towards the pain caused by inflammatory cytokines and direct peripheral nerve stimulation [50]. In this study, three targets related to pain were identified. nAChR7α was abundantly expressed in the central and peripheral nervous systems, and involved in subchronic pain and inflammation [51]. In the profiling results, nAChR7α was picked out by five alkaloids (**C2**, **C11**, **C15**, **C19**, and **C25**). MAPK p38 fished by **C9**, **C14**, and **C20** was involved in the development and maintenance of inflammatory pain [52, 53]. The reductase ALR fished by **C19** was a specific target of painful diabetic neuropathy [54, 55]. Inhibitors of ALR relieved pain and improved somatic and autonomic nerve function [56]. In addition, based on the action network, berberines (Ber) such as **C19** and **C20** may also be involved in the analgesic activity of *M. cordata*.

#### Anti-inflammatory activity

Eight targets related to inflammation were identified in this study. Phosphatidylinositol-4, 5-bisphosphate 3-kinase catalytic subunit gamma isoform (PI3 Kγ) fished by **C19** recruited leukocytes [57]. The proteinase MMP12, also known as macrophage metalloelastase (MME) or macrophage elastase (ME), was identified with three fitted compounds (**C3**, **C5**, and **C9**) in this study. MMP12 mediated neutrophil and macrophage recruitment and T cell polarization [58], and was a potential therapeutic target for asthma [59]. PPARγ* fished by **C3** was another inflammation-related target. Some early findings demonstrated the anti-inflammatory effects of PPARγ by activating human or murine monocytes/macrophages and monocyte/macrophage cell lines [60].

MAPK p38 was involved in a signaling cascade controlling cellular responses to inflammatory cytokines, and it was verified for this pathway in murine macrophage RAW264.7 cells that the *M. cordata* extract increased both the mRNA and protein levels of cytoprotective enzymes including heme oxygenase-1 (HO-1) and thioredoxin 1 via activation of the p38 MAPK/Nrf2 pathway [16]. The kinase calcium/calmodulin-dependent protein kinase II (CAMKII) was a regulator of intracellular Ca^2+^ levels, which triggered activation of the transcription factor nuclear factor-kappa B (NF-κB) after T-cell receptor stimulation. An inhibitory effect of CAMKII on NF-κB was confirmed [61]. Phospholipase A2 (PLA2s) was a key enzyme in prostaglandin (PG) biosynthesis for discharging arachidonic acid. Selective inhibitors of PLA2s were implicated in inflammation and connected to diverse diseases, such as cancer, ischemia, atherosclerosis, and schizophrenia [62].

The target mineralocorticoid receptor (MR) fished by five compounds (**C3**, **C4**, **C6**, **C7**, and **C20**) was activated by mineralocorticoids, such as aldosterone and deoxycorticosterone, as well as by glucocorticoids, like cortisol. Antagonists of MR had cardioprotective and anti-inflammatory effects in vivo via aldosterone-independent mechanisms [63]. Macrophage migration inhibitory factor (MIF) was involved in both innate and adaptive immune responses. Inhibitors of MIF were potential anti-inflammatory agents [64].

**Fig. 9:**
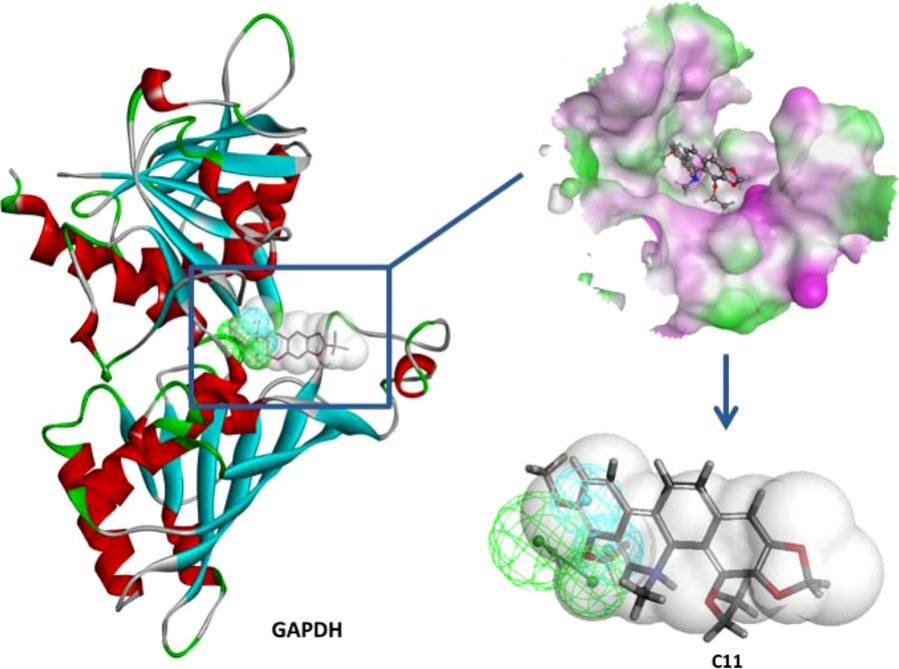
Alkaloid **C11** mapped to GAPDH. *Left* the crystal structure and pharmacophore of GAPDH, *upper right* the alkaloid **C11** docked into the target, *lower* *right* **C11** fitting into the pharmacophore and the shape of the pocket

Seven of the eight predicted targets were also related to cancer. These dual correlative targets were PI3Kγ, MMP12, PPARγ*, MAPK p38, CAMKII, PLA2s, and MIF. Their matching compounds are shown in Fig. 9, and the benzo*[c]*phenanthridine (Ben) alkaloids and berberine (Ber) alkaloids were involved in the anti-inflammatory activity.

#### Injury healing activity

In this study, four predicted targets (ERα*, ERβ*, MR, and COMP) were involved in injury repair. Among them, ERα*, ERβ*, and MR were linked with internal injuries, such as brain injury [65], vascular injury [66], and neuronal injury [67]. The other target, cartilage oligomeric matrix protein (COMP), found by **C9** was a non-collagenous extracellular matrix protein found predominantly in cartilage, but also in tendon, ligament, and meniscus [68]. COMP was a marker for joint destruction associated with osteoarthritis, rheumatoid arthritis, trauma, and intense activity [69].

#### Antitumor activity

Both the mixed and single alkaloids of *M. cordata* strongly inhibited proliferation and induced apoptosis of cancer cells [6, 70]. The anticancer drug Ukrain™ is an isoquinoline type. The major components of Ukrain™ are chelidonine, sanguinarine, chelerythrine, protopine, and allocryptopine. Ukrain™ exerted cytotoxic effects in cancer cells without negative effects on normal cells [71], and had radiosensitization effects on cancer cells, while exerting radioprotective effects on normal cells [72].

In the pharmacological profiling results, almost half of the predicted targets (31 of 65 targets) had a close relationship with cancer, and ten of them (Table 3) successfully entered into clinical trial observations. In total, nine targets related to cancer were fished by more than five compounds. The results revealed promising prospects for *M. cordata* in antitumor drug research and development. Based on the action network (Fig. 5), possible antitumor molecular mechanisms of *M.cordata* were analyzed as follows: (1) most possible effective targets and (2) most likely contributing compounds.

**Fig. 10:**
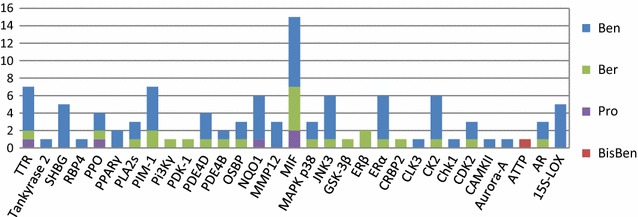
The alkaloids mapping of cancer related targets

The MIF column was particularly tall (Fig. 10) because it was fished by 15 compounds, including all quaternary benzo*[c]*phenanthridine (Ben) alkaloids (**C11–C16**), two other benzo*[c]*phenanthridine (Ben) alkaloids, five protoberberine (Ber) alkaloids, and two protopine (Pro) alkaloids. The discovered pathways of these 15 compounds mainly included NF-κB and ERK signaling pathways [73, 74], Bax/Bcl and caspase-dependent pathway [75], ROS-mediated mitochondrial pathway [76], p38 MAPK/Nrf2 pathway [77], and VEGF-induced Akt phosphorylation pathway [78]. All of these pathways were linked closely with MIF [79–84]. However, there have been no experimental reports on to the interactions between MIF and these alkaloids.

Both transthyretin (TTR) and proto-oncogene serine threonine kinase* (PIM-1) were found by seven compounds. TTR was a biomarker for lung cancer [85] and pancreatic ductal adenocarcinoma [86], but has not yet been confirmed as a therapeutic target. PIM-1* fished by **C5**, **C6**, **C8**, **C9**, **C14**, **C19**, and **C20**, and also verified by HypoDB screening, was responsible for cell cycle regulation, antiapoptotic activity, mediation of homing, and migration of receptor tyrosine kinases via the JAK/STAT pathway. PIM-1 was upregulated in many hematological malignancies and solid tumors. Although PIM kinases were described as weak oncogenes, they were heavily targeted for anticancer drug discovery [87]. **C12** was partially involved in the JAK/STAT pathway [88].

**Fig. 11:**
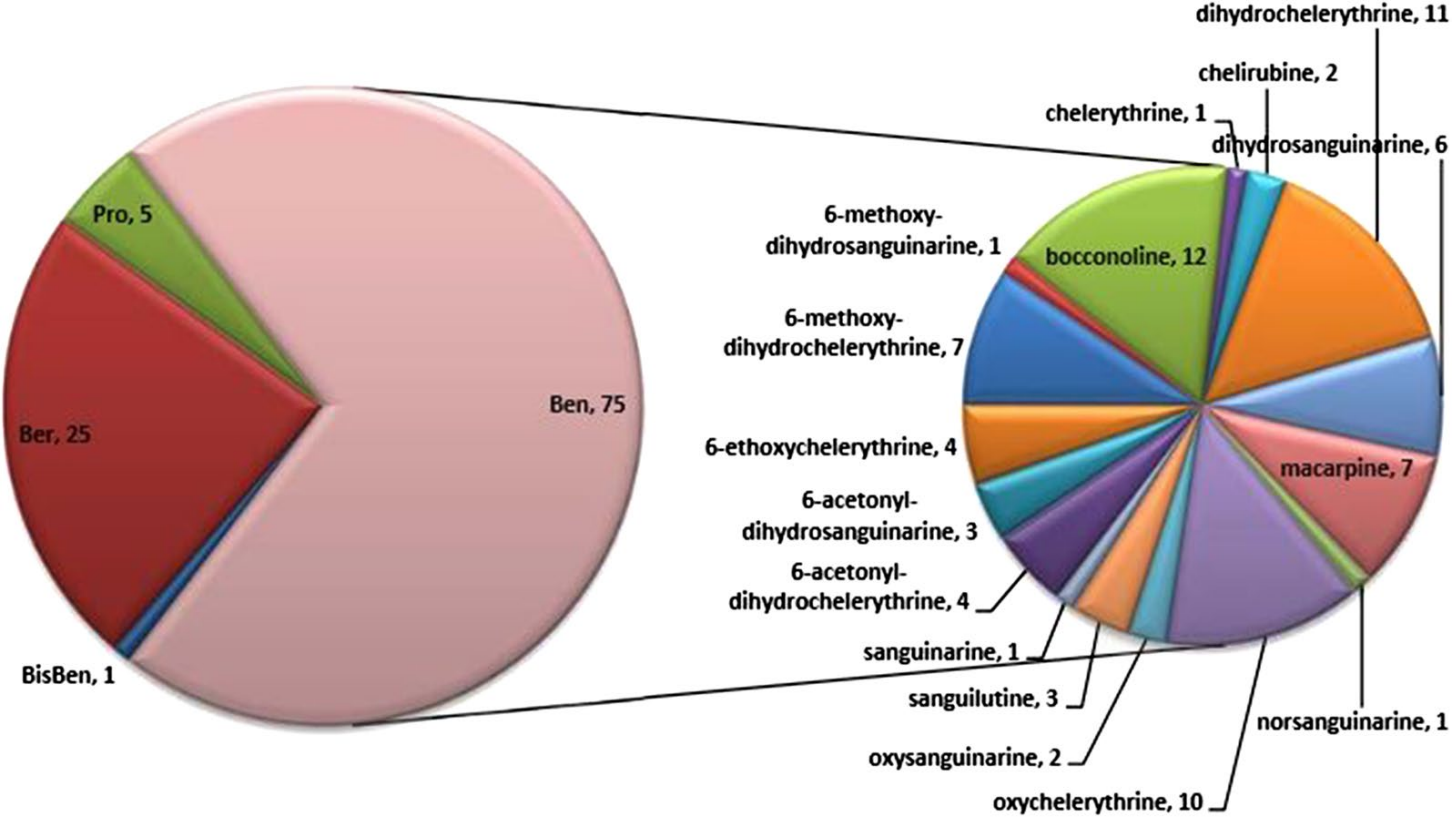
The hit number of the alkaloids to cancer related targets

The benzo*[c]*phenanthridine (Ben) alkaloids of *M. cordata* hit cancer-related targets a total of 75 times, compared with 25 times for protoberberines (Ber), five times for protopines (Pro), and one time for bis-benzo*[c]*phenanthridines (BisBen) (Fig. 11). According to the quantitative determination of alkaloids from *M. cordata*, the quaternary benzo*[c]*phenanthridine alkaloids **C12**, **C13**, and **C15** were the main active components [89]. However, the dihydro-benzo*[c]*phenanthridines such as **C5**, **C6**, and **C9** rarely reached the limit of detection (LOD), and hit more targets than the main alkaloids. As the quaternary and dihydro-benzo*[c]*phenanthridines can be transformed into one another, the dihydro-benzo*[c]*phenanthridines could be active compounds in vivo. The metabolism of **C15** was examined in pig liver microsomes and cytosol by electrospray ionization hybrid ion trap/time-of-flight mass spectrometry, and **C7** was one of the main metabolites in liver microsomes and the only metabolite in cytosol [90]. Hence, the issue of whether the dihydro-benzo*[c]*phenanthridines were the main compounds combining with the targets in vivo requires further investigation.

Among the 31 cancer-related targets, at least seven (including MIF, PPARγ*, CAMKII, and Pi3Kγ) were involved in the immune system. These immune-associated targets might be crucial to for oncotherapy with *M. cordata.*

### Potential pharmacological activities

According to the pharmacological profiling, some unreported pharmacological performances of *M.cordata* emerged. In this study, 10 targets linked with neurodegeneration were fished, among which AChE and MAO-B were crucial therapeutic targets in Alzheimer’s disease and Parkinson’s disease [91–94].

In addition, antiviral activities, especially anti-HIV, anti-SARS coronavirus, and antifungal activities, were kinds of extensions of the antibacterial function of *M. cordata*. The possible anti-HIV activity was notable, because HIV-1 reverse transcriptase and multidrug-resistant HIV-1 protease* were particularly related to AIDS [95–99]. Meanwhile, the anti-HIV activity was partly confirmed by HypoDB screening. The protein SARS-CoV M(pro) predicted by **C3** and **C5** was an attractive target for structure-based drug design of anti-SARS drugs owing to its indispensability for the maturation of severe acute respiratory syndrome coronavirus (SARS-CoV) [100]. Another target, HS5B Pol, fished by five alkaloids was a target for anti-HCV therapeutic advances [101]. Inhibitors of HS5B Pol would be a principal option for the treatment of HCV [102]. Meanwhile, scytalone dehydratase and negative transcriptional regulator NmrA were suggested to be physiological targets of new fungicides and the subjects of inhibitor design and optimization [103–105].

In this paper, we proposed a very wide range of the promising targets for the isoquinoline alkaloids of *M. cordata*. Most of the hits are not yet proven by pharmacological experiment.

## Conclusion

Through in silicotarget fishing, the anticancer, anti-inflammatory, and analgesic effects of *M. cordata* were the most significant among many possible activities. The possible anticancer effects were mainly contributed by the isoquinoline alkaloids as active components.

## Abbreviations

CHARMM: chemistry at Harvard Macromolecular Mechanics
MOE: molecular operating environment
RMSD: root mean square deviation
MMFF: Merck molecular force field
PDB: Protein Data Bank
KEGG: Kyoto Encyclopedia of genes and genomes
TTD: Therapeutic Target Database
HTML: hypertext markup language

## References

[1] Xu GJ, Wang Q, Yu BM. Color illustrations of antitumor traditional Chinese medicine. Fuzhou: Fujian Sci Technol Publ House; 1997. p. 759.

[2] Grieve M. A modern herbal. Middlesex: Penguin Books; 1984.

[3] Duke JA, Ayenus ES. Medicinal plants of China. Inc. Algonac: Reference Publications; 1984.

[4] Psotova J, Vecera R, Zdarilova A, Anzenbacherova E, Kosina P, Svobodova A, Hrbac J (2006). Safety assessment of sanguiritrin, alkaloid fraction of *Macleaya cordata*, in rats. Vet Med-Czech.

[5] Pang JX, Ma RQ, Liu LM, Jiang YP, Sun LS (2005). In vitro cytotoxic effect on Hep3B cells and in vivo antitumor effect in mice. J First Mil Med Univ.

[6] Yang S, Liu Y, Yang QF, Xiang JF, Tang YL, Xu GZ (2011). Antitumor effect of *Macleaya cordata* and its molecular mechanism on inducement of human telomeric DNA to form G-quadruplex. Chin Trad Herb Drugs.

[7] Pang FG. Study on the Anticancer Constituents of *Macleaya cordata* (Willd) R.Br. Master Thesis. Shenyang Pharmaceutical University; 2005.

[8] Kemeny-Beke A, Aradi J, Damjanovich J, Beck Z, Facsko A, Berta A, Bodnar A (2006). Apoptotic response of uveal melanoma cells upon treatment with chelidonine, sanguinarine and chelerythrine. Cancer Lett.

[9] Lenfeld J, Kroutil M, Marsálek E, Slavik J, Preininger V, Simánek V (1981). Antiinflammatory activity of quaternary benzophenanthridine alkaloids from *Chelidonium majus*. Planta Med.

[10] Park JE, Cuong TD, Hung TM, Lee I, Na M, Kim JC, Ryoo S, Lee JH, Choi JS, Woo MH (2011). Alkaloids from *Chelidonium majus* and their inhibitory effects on LPS-induced NO production in RAW264.7 cells. Bioorg Med Chem Lett.

[11] Xiao L, Yi J, Zhao J, Xu L, Liu BY, Liu DM, Zeng JG (2011). Protective effect of *Macleaya cordata* extract on alcohol-induced acute hepatic injury in rats. Cent South Pharm.

[12] Kosina P, Gregorova J, Gruz J, Vacek J, Kolar M, Vogel M, Roos W, Naumann K, Simanek V, Ulrichova J (2010). Phytochemical and antimicrobial characterization of *Macleaya cordata* herb. Fitoterapia.

[13] Cheng RB, Chen X, Liu SJ, Zhang GH (2007). Effect of Chelerythrine on glucosyltransferase and water-insoluble glucan of *Streptococcus mutans*. Shanghai J Stomatol.

[14] Yu JP, Zhao DL, Meng XB, Zhou XQ (2006). The antibacterial effect of the alkaloids from *Macleaya cordata* on eight kinds of fungi. J Mount Agri Biol.

[15] Hiller KO, Ghorbani M, Schilcher H (1998). Antispasmodic and relaxant activity of chelidonine, protopine, coptisine, and *Chelidonium majus* extracts on isolated guinea-pig ileum. Planta Med.

[16] Vrba J, Orolinova E, Ulrichova J (2012). Induction of heme oxygenase-1 by *Macleaya cordata* extract and its constituent sanguinarine in RAW264.7 cells. Fitoterapia.

[17] Lei QF, Zhao XL, Xu LJ, Peng Y, Xiao PG (2014). Chemical constituents of plants from tribe Chelidonieae and their bioactivities. Chin Herb Med.

[18] Liu LJ, Leung KH, Lin S, Chan DS, Susanti D, Rao W, Chan PW, Ma DL, Leung CH (2015). Pharmacophore modeling for the identification of small-molecule inhibitors of TACE. Methods.

[19] Zhong HJ, Liu LJ, Chong CM, Lu L, Wang M, Chan DS, Chan PW, Lee SM, Ma DL, Leung CH (2014). Discovery of a natural product-like iNOS inhibitor by molecular docking with potential neuroprotective effects in vivo. PLoS One.

[20] Ma DL, Chan DS, Wei G, Zhong HJ, Yang H, Leung LT, Gullen EA, Chiu P, Cheng YC, Leung CH (2014). Virtual screening and optimization of Type II inhibitors of JAK2 from a natural product library. Chem Commun (Camb).

[21] Li H, Gao Z, Kang L, Zhang H, Yang K, Yu K, Luo X, Zhu W, Chen K, Shen J (2006). TarFisDock: a web server for identifying drug targets with docking approach. Nucleic Acids Res.

[22] Schuster D (2010). 3D pharmacophores as tools for activity profiling. Drug Discov Today Tech.

[23] Steindl TM, Schuster D, Laggner C, Langer T (2006). Parallel screening: a novel concept in pharmacophore modelling and virtual screening. J Chem Inf Model.

[24] Steindl TM, Schuster D, Wolber G, Laggner C, Langer T (2006). High throughput structure-based pharmacophore modeling as a basis for successful parallel virtual screening. J Comput Aided Mol Des.

[25] Meslamani J, Li J, Sutter J, Stevens A, Bertrand HO, Rognan D (2012). Protein–ligand-based pharmacophores: generation and utility assessment in computational ligand profiling. J Chem Inf Model.

[26] Meslamani J, Rognan D, Kellenberger E (2011). sc-PDB: a database for identifying variations and multiplicity of ‘druggable’ binding sites in proteins. Bioinformatics.

[27] Zhu F, Shi Z, Qin C, Tao L, Liu X, Xu F, Zhang L, Song Y, Liu X, Zhang J (2012). Therapeutic target database update 2012: a resource for facilitating target-oriented drug discovery. Nucleic Acids Res.

[28] Knox C, Law V, Jewison T, Liu P, Ly S, Frolkis A, Pon A, Banco K, Mak C, Neveu V (2011). DrugBank 3.0: a comprehensive resource for ‘omics’ research on drugs. Nucleic Acids Res.

[29] Smoot ME, Ono K, Ruscheinski J, Wang PL, Ideker T (2011). Cytoscape 2.8: new features for data integration and network visualization. Bioinformatics.

[30] Ehrman TM, Barlow DJ, Hylands PJ (2010). In silico search for multi-target anti-inflammatories in Chinese herbs and formulas. Bioorg Med Chem.

[31] Li B, Xu X, Wang X, Yu H, Li X, Tao W, Wang Y, Yang L (2012). A systems biology approach to understanding the mechanisms of action of Chinese herbs for treatment of cardiovascular disease. Int J Mol Sci.

[32] Li X, Xu X, Wang J, Yu H, Wang X, Yang H, Xu H, Tang S, Li Y, Yang L (2012). A system-level investigation into the mechanisms of Chinese Traditional Medicine: compound Danshen formula for cardiovascular disease treatment. PLoS One.

[33] Zhao DL, Yu JP, Zhou XQ (2005). Antibacterial effect of the Sanguinarine Hydrochloride and Bocconoline from *Macleaya cordata*. Food Sci.

[34] Fibriansah G, Kovacs AT, Pool TJ, Boonstra M, Kuipers OP, Thunnissen AM (2012). Crystal structures of two transcriptional regulators from *Bacillus cereus* define the conserved structural features of a PadR subfamily. PLoS One.

[35] Agustiandari H, Peeters E, de Wit JG, Charlier D, Driessen AJ (2011). LmrR-mediated gene regulation of multidrug resistance in *Lactococcus lactis*. Microbiology.

[36] Salverda ML, De Visser JA, Barlow M (2010). Natural evolution of TEM-1 beta-lactamase: experimental reconstruction and clinical relevance. FEMS Microbiol Rev.

[37] Ehmann DE, Jahic H, Ross PL, Gu RF, Hu J, Kern G, Walkup GK, Fisher SL (2012). Avibactam is a covalent, reversible, non-beta-lactam beta-lactamase inhibitor. Proc Natl Acad Sci USA.

[38] Shaw WV (1983). Chloramphenicol acetyltransferase: enzymology and molecular biology. CRC Crit Rev Biochem.

[39] Catalano-Dupuy DL, Lopez-Rivero A, Soldano A, Ceccarelli EA (2013). Redox proteins as targets for drugs development against pathogens. Curr Pharm Des.

[40] Willems AR, Tahlan K, Taguchi T, Zhang K, Lee ZZ, Ichinose K, Junop MS, Nodwell JR (2008). Crystal structures of the *Streptomyces coelicolor*TetR-like protein ActR alone and in complex with actinorhodin or the actinorhodin biosynthetic precursor (S)-DNPA. J Mol Biol.

[41] Tsao DH, Sutherland AG, Jennings LD, Li Y, Rush TR, Alvarez JC, Ding W, Dushin EG, Dushin RG, Haney SA (2006). Discovery of novel inhibitors of the ZipA/FtsZ complex by NMR fragment screening coupled with structure-based design. Bioorg Med Chem.

[42] Ling LL, Xian J, Ali S, Geng B, Fan J, Mills DM, Arvanites AC, Orgueira H, Ashwell MA, Carmel G (2004). Identification and characterization of inhibitors of bacterial enoyl-acyl carrier protein reductase. Antimicrob Agents Chemother.

[43] Yao JY, Shen JY, Li XL, Xu Y, Hao GJ, Pan XY, Wang GX, Yin WL (2010). Effect of sanguinarine from the leaves of *Macleaya cordata* against *Ichthyophthirius multifiliis* in grass carp (*Ctenopharyngodon idella*). Parasitol Res.

[44] Yao JY, Zhou ZM, Li XL, Yin WL, Ru HS, Pan XY, Hao GJ, Xu Y, Shen JY (2011). Antiparasitic efficacy of dihydrosanguinarine and dihydrochelerythrine from *Macleaya microcarpa* against *Ichthyophthirius multifiliis* in richadsin (*Squaliobarbus curriculus*). Vet Parasitol.

[45] Wang GX, Zhou Z, Jiang DX, Han J, Wang JF, Zhao LW, Li J (2010). In vivo anthelmintic activity of five alkaloids from *Macleaya microcarpa* (Maxim) Fedde against *Dactylogyru sintermedius* in Carassiusauratus. Vet Parasitol.

[46] Juskiewicz J, Gruzauskas R, Zdunczyk Z, Semaskaite A, Jankowski J, Totilas Z, Jarule V, Sasyte V, Zdunczyk P (2011). Raceviciute-StupelieneA: effects of dietary addition of *Macleaya cordata* alkaloid extract on growth performance, caecal indices and breast meat fatty acids profile in male broilers. J Anim Physiol Anim Nutr (Berl).

[47] Sarma GN, Savvides SN, Becker K, Schirmer M, Schirmer RH, Karplus PA (2003). Glutathione reductase of the malarial parasite *Plasmodium falciparum*: crystal structure and inhibitor development. J Mol Biol.

[48] Leitão A, Andricopulo AD, Oliva G, Pupo MT, de Marchi AA, Vieira PC, da Silva MF, Ferreira VF, de Souza MC, Sá MM, Moraes VR, Montanari CA (2004). Structure–activity relationships of novel inhibitors of glyceraldehyde-3-phosphate dehydrogenase. Bioorg Med Chem Lett.

[49] Pinheiro MP, Emery FS, Nonato MC (2013). Target sites for the design of anti-trypanosomatid drugs based on the structure of dihydroorotatedehydrogenase. Curr Pharm Des.

[50] Cai YT, Ju CY, Yan HR, Sun LQ (2011). Experimental research on analgesic effect of the total alkaloid from Celandine. J Mudanjiang Med Univ.

[51] Freitas K, Carroll FI, Damaj MI (2013). The antinociceptive effects of nicotinic receptors alpha7-positive allosteric modulators in murine acute and tonic pain models. J Pharmacol Exp Ther.

[52] Medicherla S, Reddy M, Ying J, Navas TA, Li L, Nguyen AN, Kerr I, Hanjarappa N, Protter AA, Higgins LS (2008). p38alpha-selective MAP kinase inhibitor reduces tumor growth in mouse xenograft models of multiple myeloma. Anticancer Res.

[53] Laufer S, Lehmann F (2009). Investigations of SCIO-469-like compounds for the inhibition of p38 MAP kinase. Bioorg Med Chem Lett.

[54] Steuber H, Heine A, Klebe G (2007). Structural and thermodynamic study on aldose reductase: nitro-substituted inhibitors with strong enthalpic binding contribution. J Mol Biol.

[55] Suryanarayana P, Kumar PA, Saraswat M, Petrash JM, Reddy GB (2004). Inhibition of aldose reductase by tannoid principles of *Emblica officinalis*: implications for the prevention of sugar cataract. Mol Vis.

[56] Young RJ, Ewing DJ, Clarke BF (1983). A controlled trial of sorbinil, an aldose reductase inhibitor, in chronic painful diabetic neuropathy. Diabetes.

[57] Wymann MP, Solinas G (2013). Inhibition of phosphoinositide 3-kinase gamma attenuates inflammation, obesity, and cardiovascular risk factors. Ann N Y Acad Sci.

[58] Dufour A, Overall CM (2013). Missing the target: matrix metalloproteinase antitargets in inflammation and cancer. Trends Pharmacol Sci.

[59] Mukhopadhyay S, Sypek J, Tavendale R, Gartner U, Winter J, Li W, Page K, Fleming M, Brady J, O’Toole M (2010). Matrix metalloproteinase-12 is a therapeutic target for asthma in children and young adults. J Allergy Clin Immunol.

[60] Clark RB (2002). The role of PPARs in inflammation and immunity. J Leukoc Biol.

[61] Maubach G, Sokolova O, Wolfien M, Rothkotter HJ, Naumann M (2013). Ca/calmodulin-dependent kinase II contributes to inhibitor of nuclear factor-kappa B kinase complex activation in *Helicobacter pylori* infection. Int J Cancer.

[62] Mahalka AK, Kinnunen PK (2013). Class specific peptide inhibitors for secretory phospholipases A2. Biochem Biophys Res Commun.

[63] Usher MG, Duan SZ, Ivaschenko CY, Frieler RA, Berger S, Schutz G, Lumeng CN, Mortensen RM (2010). Myeloid mineralocorticoid receptor controls macrophage polarization and cardiovascular hypertrophy and remodeling in mice. J Clin Invest.

[64] Xu L, Li Y, Sun H, Zhen X, Qiao C, Tian S, Hou T (2013). Current developments of macrophage migration inhibitory factor (MIF) inhibitors. Drug Discov Today.

[65] Dubal DB, Zhu H, Yu J, Rau SW, Shughrue PJ, Merchenthaler I, Kindy MS, Wise PM (2001). Estrogen receptor alpha, not beta, is a critical link in estradiol-mediated protection against brain injury. Proc Natl Acad Sci USA.

[66] Karas RH, Hodgin JB, Kwoun M, Krege JH, Aronovitz M, Mackey W, Gustafsson JA, Korach KS, Smithies O, Mendelsohn ME (1999). Estrogen inhibits the vascular injury response in estrogen receptor beta-deficient female mice. Proc Natl Acad Sci USA.

[67] Macleod MR, Johansson IM, Soderstrom I, Lai M, Gido G, Wieloch T, Seckl JR, Olsson T (2003). Mineralocorticoid receptor expression and increased survival following neuronal injury. Eur J Neurosci.

[68] Smith RK, Heinegard D (2000). Cartilage oligomeric matrix protein (COMP) levels in digital sheath synovial fluid and serum with tendon injury. Equine Vet J.

[69] Posey KL, Hecht JT (2008). The role of cartilage oligomeric matrix protein (COMP) in skeletal disease. Curr Drug Targets.

[70] Fan SL, Jiao F, Zhang Y, An CX, Fu JM (2000). Study on effect of total alkaloids of *Macleaya cordata* to animal’s transplanted tumor. Shanxi Onco Med.

[71] Hohenwarter O, Strutzenberger K, Katinger H, Liepins A, Nowicky JW (1992). Selective inhibition of in vitro cell growth by the anti-tumour drug Ukrain. Drugs Exp Clin Res.

[72] Cordes N, Plasswilm L, Bamberg M, Rodemann HP (2002). Ukrain, an alkaloid thiophosphoric acid derivative of *Chelidonium majus* L. protects human fibroblasts but not human tumour cells in vitro against ionizing radiation. Int J Radiat Biol.

[73] Li H, Zhai Z, Liu G, Tang T, Lin Z, Zheng M, Qin A, Dai K (2013). Sanguinarine inhibits osteoclast formation and bone resorption via suppressing RANKL-induced activation of NF-kappaB and ERK signaling pathways. Biochem Biophys Res Commun.

[74] Ansari KM, Das M (2010). Skin tumor promotion by argemone oil/alkaloid in mice: evidence for enhanced cell proliferation, ornithine decarboxylase, cyclooxygenase-2 and activation of MAPK/NF-kappaB pathway. Food Chem Toxicol.

[75] Lee JS, Jung WK, Jeong MH, Yoon TR, Kim HK (2012). Sanguinarine induces apoptosis of HT-29 human colon cancer cells via the regulation of Bax/Bcl-2 ratio and caspase-9-dependent pathway. Int J Toxicol.

[76] Choi WY, Kim GY, Lee WH, Choi YH (2008). Sanguinarine, a benzophenanthridine alkaloid, induces apoptosis in MDA-MB-231 human breast carcinoma cells through a reactive oxygen species-mediated mitochondrial pathway. Chemotherapy.

[77] Vrba J, Orolinova E, Ulrichova J (2012). Induction of heme oxygenase-1 by *Macleaya cordata* extract and its constituent sanguinarine in RAW264.7 cells. Fitoterapia.

[78] Basini G, Santini SE, Bussolati S, Grasselli F (2007). Sanguinarine inhibits VEGF-induced Akt phosphorylation. Ann N Y Acad Sci.

[79] Hussain F, Freissmuth M, Volkel D, Thiele M, Douillard P, Antoine G, Thurner P, Ehrlich H, Schwarz HP, Scheiflinger F (2013). Human anti-macrophage migration inhibitory factor (MIF) antibodies inhibit growth of human prostate cancer cells in vitro and in vivo. Mol Cancer Ther.

[80] Guo Y, Hou J, Luo Y, Wang D (2013). Functional disruption of macrophage migration inhibitory factor (MIF) suppresses proliferation of human H460 lung cancer cells by caspase-dependent apoptosis. Cancer Cell Int.

[81] Wadgaonkar R, Somnay K, Garcia JG (2008). Thrombin induced secretion of macrophage migration inhibitory factor (MIF) and its effect on nuclear signaling in endothelium. J Cell Biochem.

[82] Sun B, Nishihira J, Suzuki M, Fukushima N, Ishibashi T, Kondo M, Sato Y, Todo S (2003). Induction of macrophage migration inhibitory factor by lysophosphatidic acid: relevance to tumor growth and angiogenesis. Int J Mol Med.

[83] Chuang YC, Su WH, Lei HY, Lin YS, Liu HS, Chang CP, Yeh TM (2012). Macrophage migration inhibitory factor induces autophagy via reactive oxygen species generation. PLoS One.

[84] Mathew B, Jacobson JR, Siegler JH, Moitra J, Blasco M, Xie L, Unzueta C, Zhou T, Evenoski C, Al-Sakka M (2013). Role of migratory inhibition factor in age-related susceptibility to radiation lung injury via NF-E2-related factor-2 and antioxidant regulation. Am J Respir Cell Mol Biol.

[85] Liu L, Sun S, Liu J, Wu S, Dai S, Wang X, Huang L, Xiao X, He D (2009). A new serum biomarker for lung cancer—transthyretin. Zhongguo Fei Ai Za Zhi.

[86] Chen J, Chen LJ, Xia YL, Zhou HC, Yang RB, Wu W, Lu Y, Hu LW, Zhao Y (2013). Identification and verification of transthyretin as a potential biomarker for pancreatic ductal adenocarcinoma. J Cancer Res Clin Oncol.

[87] Blanco-Aparicio C, Carnero A (2013). Pim kinases in cancer: diagnostic, prognostic and treatment opportunities. Biochem Pharmacol.

[88] Pan J, Fukuda K, Saito M, Matsuzaki J, Kodama H, Sano M, Takahashi T, Kato T, Ogawa S (1999). Mechanical stretch activates the JAK/STAT pathway in rat cardiomyocytes. Circ Res.

[89] Pencikova K, Urbanova J, Musil P, Taborska E, Gregorova J (2011). Seasonal variation of bioactive alkaloid contents in *Macleaya microcarpa* (Maxim.) Fedde. Molecules.

[90] Zhang HH, Wu Y, Sun ZL, Liu ZY (2013). Identification of sanguinarine metabolites in pig liver preparations by accurate mass measurements using electrospray ionization hybrid ion trap/time-of-flight mass spectrometry. Rapid Commun Mass Spectrum.

[91] Fang L, Gou S, Fang X, Cheng L, Fleck C (2013). Current progresses of novel natural products and their derivatives/analogs as anti-Alzheimer candidates: an update. Mini Rev Med Chem.

[92] Greenfield S, Vaux DJ (2002). Parkinson’s disease, Alzheimer’s disease and motor neurone disease: identifying a common mechanism. Neurosci.

[93] Huang L, Lu C, Sun Y, Mao F, Luo Z, Su T, Jiang H, Shan W, Li X (2012). Multitarget-directed benzylideneindanone derivatives: anti-beta-amyloid (Abeta) aggregation, antioxidant, metal chelation, and monoamine oxidase B (MAO-B) inhibition properties against Alzheimer’s disease. J Med Chem.

[94] DeMarcaida JA, Schwid SR, White WB, Blindauer K, Fahn S, Kieburtz K, Stern M, Shoulson I (2006). Effects of tyramine administration in Parkinson’s disease patients treated with selective MAO-B inhibitor rasagiline. Mov Disord.

[95] Boyer PL, Clark PK, Hughes SH (2012). HIV-1 and HIV-2 reverse transcriptases: different mechanisms of resistance to nucleoside reverse transcriptase inhibitors. J Virol.

[96] Klarmann GJ, Hawkins ME, Le Grice SF (2002). Uncovering the complexities of retroviral ribonuclease H reveals its potential as a therapeutic target. AIDS Rev.

[97] Ambrose Z, Herman BD, Sheen CW, Zelina S, Moore KL, Tachedjian G, Nissley DV, Sluis-Cremer N (2009). The human immunodeficiency virus type 1 nonnucleoside reverse transcriptase inhibitor resistance mutation I132M confers hypersensitivity to nucleoside analogs. J Virol.

[98] Sadiq SK, Noe F, De Fabritiis G (2012). Kinetic characterization of the critical step in HIV-1 protease maturation. Proc Natl Acad Sci USA.

[99] Martin P, Vickrey JF, Proteasa G, Jimenez YL, Wawrzak Z, Winters MA, Merigan TC, Kovari LC (2005). “Wide-open” 1.3 A structure of a multidrug-resistant HIV-1 protease as a drug target. Structure.

[100] Lu IL, Mahindroo N, Liang PH, Peng YH, Kuo CJ, Tsai KC, Hsieh HP, Chao YS, Wu SY (2006). Structure-based drug design and structural biology study of novel nonpeptide inhibitors of severe acute respiratory syndrome coronavirus main protease. J Med Chem.

[101] Patel BA, Krishnan R, Khadtare N, Gurukumar KR, Basu A, Arora P, Bhatt A, Patel MR, Dana D, Kumar S (2013). Design and synthesis of ʟ- and d-phenylalanine derived rhodanines with novel C5-arylidenes as inhibitors of HCV NS5B polymerase. Bioorg Med Chem.

[102] Varshney J, Sharma PK, Sharma A (2012). A review on an update of NS5B polymerase hepatitis C virus inhibitors. Eur Rev Med Pharmacol Sci.

[103] Jordan DB, Basarab GS, Steffens JJ, Schwartz RS, Doughty JG (2000). Tight binding inhibitors of scytalonedehydratase: effects of site-directed mutations. Biochemistry-Us.

[104] Stammers DK, Ren J, Leslie K, Nichols CE, Lamb HK, Cocklin S, Dodds A, Hawkins AR (2001). The structure of the negative transcriptional regulator NmrA reveals a structural superfamily which includes the short-chain dehydrogenase/reductases. EMBO J.

[105] Zhao X, Hume SL, Johnson C, Thompson P, Huang J, Gray J, Lamb HK, Hawkins AR (2010). The transcription repressor NmrA is subject to proteolysis by three *Aspergillus nidulans* proteases. Protein Sci.

